# Supplementary material for: Structure of the DNA-Bound Spacer Capture Complex of a Type II CRISPR-Cas System
Source: Mol Cell. 2019 Jul 11;75(1):90–101.e5. doi: 10.1016/j.molcel.2019.04.020 (PMC6620040; doi:10.1016/j.molcel.2019.04.020)
Supplement: Document S1. Figures S1–S0037 [file mmc1.pdf]

Molecular Cell, Volume *75*

Supplemental Information

Structure of the DNA-Bound Spacer Capture Complex of a Type II CRISPR-Cas System

Martin Wilkinson, Gediminas Drabavicius, Arunas Silanskas, Giedrius Gasiunas, Virginijus Siksnys, and Dale B. Wigley

Figure S1: Cryo-EM images of the mixture. Related to Figure 1, Figure 2 and Figure 5.

Figure S2: EM processing for the Cas1Cas2Csn2 monomer complex. Related to Figure 2.

Figure S3: EM processing for the Cas1Cas2Csn2 dimer complex. Related to Figure 5.

Figure S4: DNA binds within a positive channel through the Csn2 tetramers. Related to Figure 2.

Figure S5: Deep-sequencing analysis of DNA captured by Cas1- Cas2-Csn2-Cas9 complex and integration assays. Related to Figure 1, Figure 2 and Figure 5.

Figure S6: *In vitro* interactions of CRISPR3-Cas proteins. Related to Figure 1.

Figure S7: EM processing for the Cas1Cas2Csn2 filament complex. Related to Figure 5.

Figure S1. Related to Figure 1, Figure 2 and Figure 5.

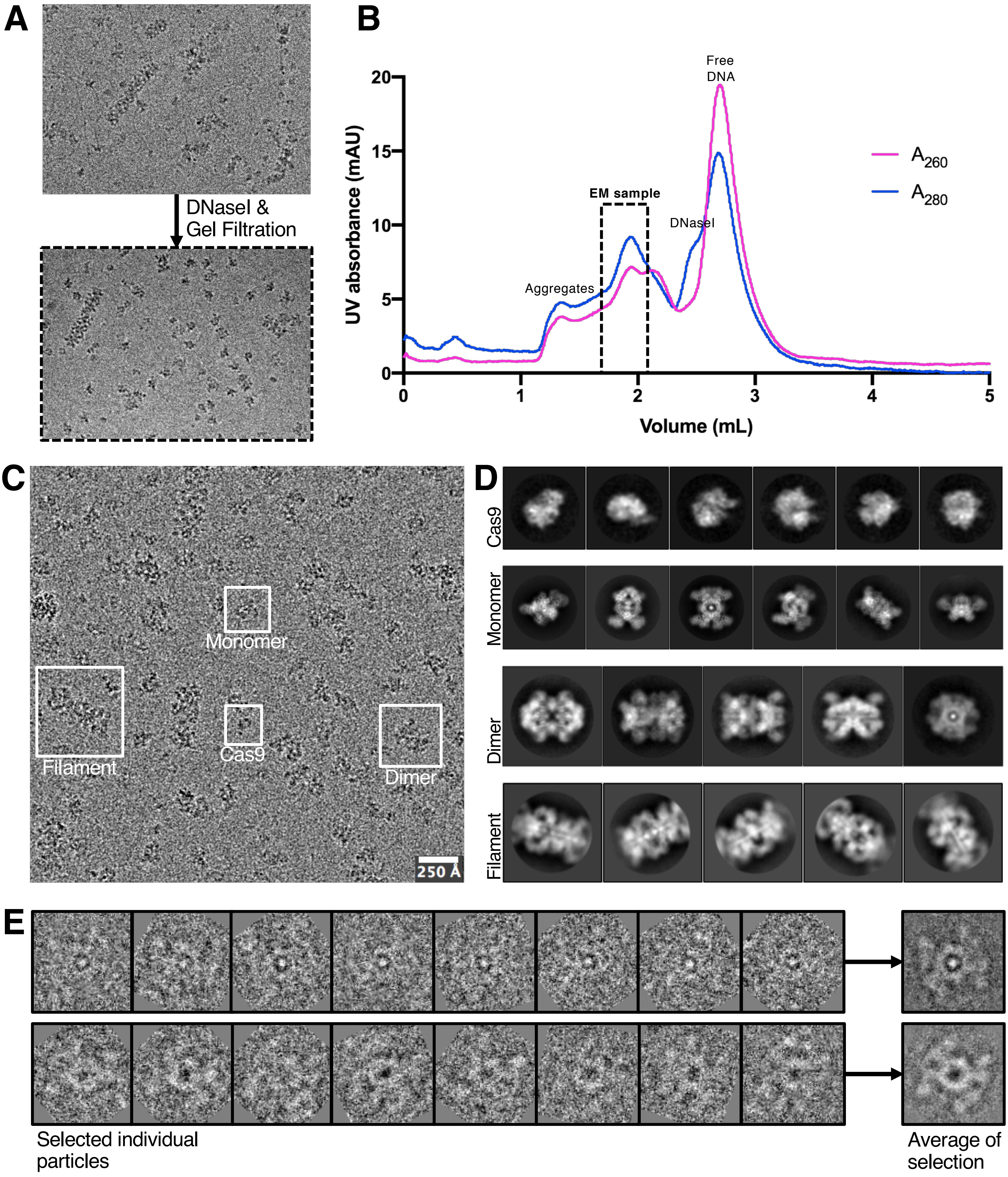

Figure S1. Cryo-EM images of the mixture. Related to Figure 1, Figure 2 and Figure 5.

(A) Initial cryo image of the sample showing long threads of DNA.

(B) Cryo image after DNaseI treatment. Both images were taken on a Technai T12 microscope with CCD detector.

(C) Section of a micrograph from the Krios-Falcon III dataset. An example of each of the different particle populations is boxed.

(D) Representative 2D classes generated in RELION from the Krios-Falcon III dataset showing different views for each of the four particle populations.

(E) A selection of particles from a subset of the Krios-Falcon III dataset that represented just the front view of the monomer complex. The particles could be separated into two species: 98.6 % were DNA bound (top row) and 1.4 % were fully assembled without DNA (bottom row). Particles and class averages were generated and displayed using Relion.

Figure S2. Related to Figure 2.

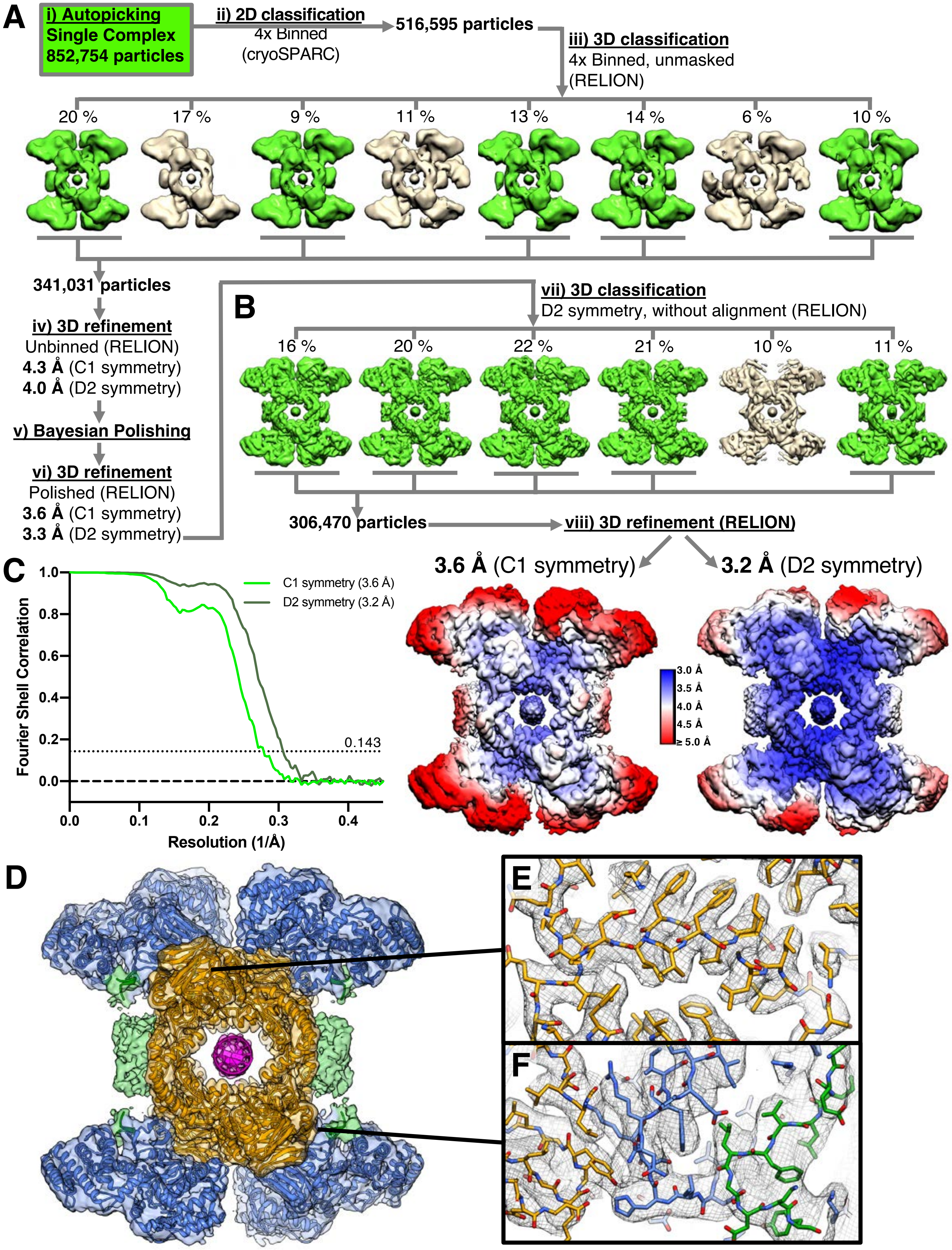

Figure S2. EM processing for the Cas1Cas2Csn2 monomer complex. Related to Figure 2.

(A) Scheme overview of the initial processing steps for the monomer particle set.

(B) The 3D classes from the final classification step are shown. The classes show flexibility in the orientation of the Cas1 dimers relative to the rest of the complex.

(C) FSC plot for the final monomer maps (left). The deposited C1 and supplemental D2-symmetrised maps are shown coloured by local resolution (calculated using RELION) (right).

(D) The deposited 3.6 Å monomer map is shown as a transparent surface with the coordinates underneath. Both are coloured similarly to Figure 2.

(E) A section of the 3.2 Å D2-symmetrised map displayed as grey mesh showing ordered side-chain density for the selected region within Csn2.

(F) A section of the 3.2 Å D2-symmetrised map displayed as grey mesh showing the fit of the contact regions between the C-terminus of Cas2, the Cas1 NTD and the Csn2 head domain.

Figure S3. Related to Figure 5.

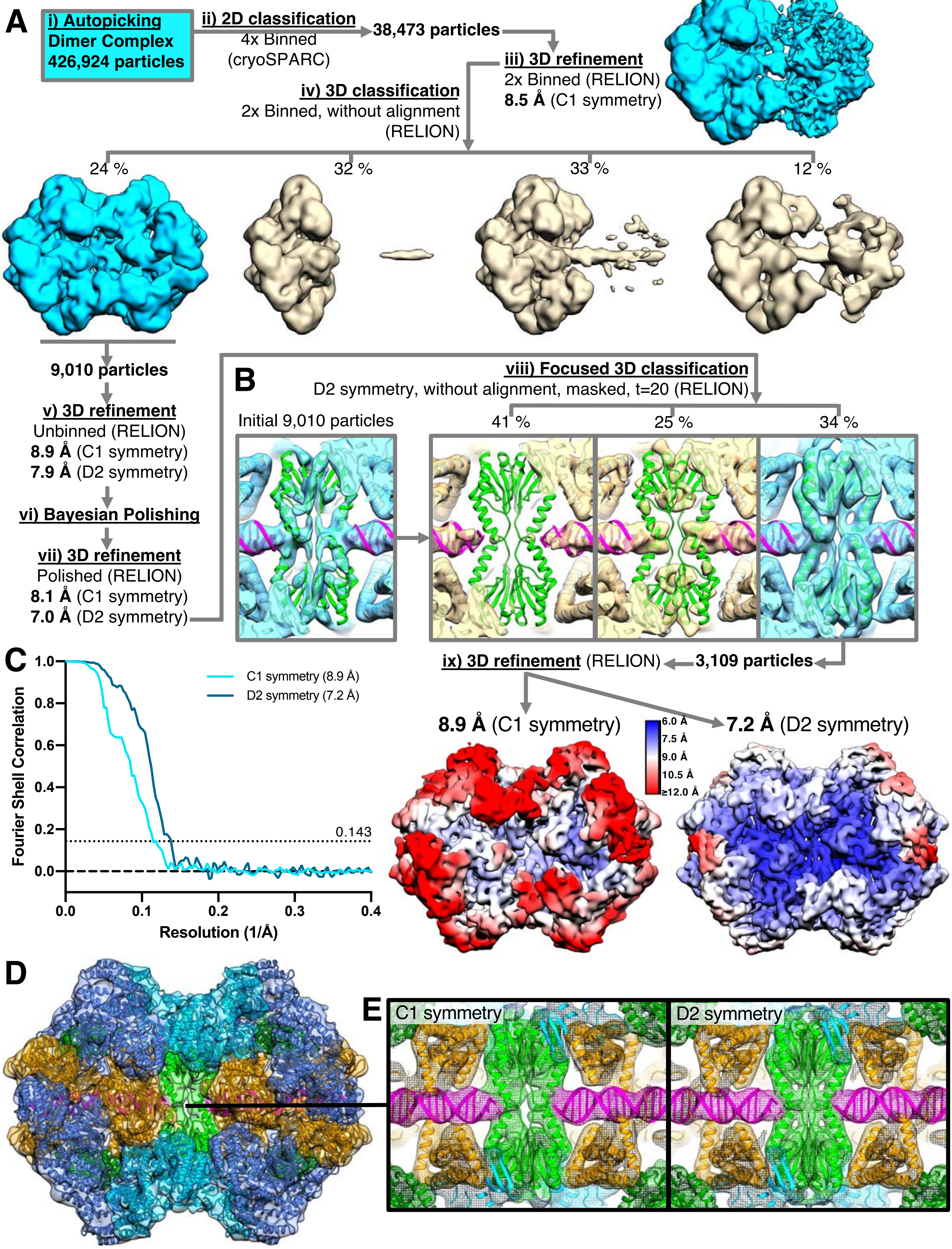

Figure S3. EM processing for the Cas1Cas2Csn2 dimer complex. Related to Figure 5.

(A) Scheme overview of the initial processing steps for the dimer particle set.

(B) A section around the middle Cas2 dimer,s which cap the DNA fragment ends, are shown for each of the 3D classes from the final focused classification step as well as for the initial map before classification. The classes show flexibility in the orientation of the Cas1 dimers relative to the rest of the complex. A homogeneous class with strong density for the region was selected for the final refinement.

(C) FSC plot for the final dimer maps (left). The deposited C1 and supplemental D2-symmetrised maps are shown coloured by local resolution (calculated using RELION) (right).

(D) The deposited 8.9 Å dimer map is shown as a transparent surface with the coordinates underneath. Both are coloured similarly to Figure 5.

(E) A section of both the C1 and D2-symmetrised maps is displayed as coloured mesh showing the fit of the ordered Cas2 dimers capping the end of the DNA fragments. There is no free density to suggest that the DNA ends are joined.

Figure S4. Related to Figure 2.

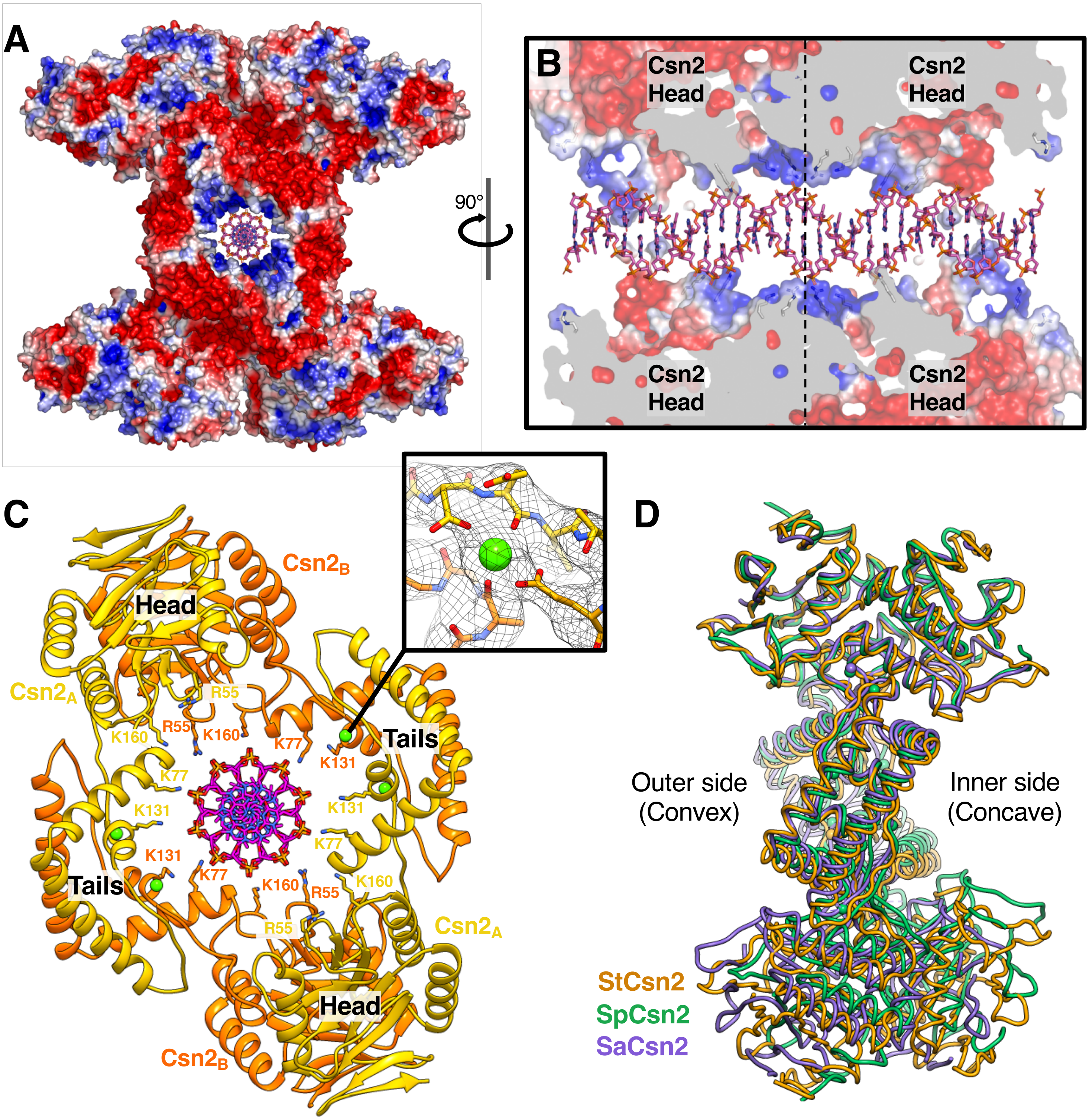

Figure S4. DNA binds within a positive channel through the Csn2 tetramers. Related to Figure 2.

(A) Overall charge distribution on the surface of the monomer structure calculated by APBS displaying a range from -3 (red) to +3 (blue) kT/e. The bound DNA was deleted from the charge calculation but overlaid for reference.

(B) Cutaway showing the charge distribution along the central channel through the two Csn2 tetramers, which are divided here by a dotted line. Due to the concave nature of the interacting Csn2 tetramer surfaces, more DNA contacts are found in the centre of the channel than in the peripheries.

(C) A single Csn2 tetramer is displayed as cartoon coloured by chain showing the conserved residues positioned to bind non-specifically to the DNA backbone. Bound Ca2+ ions are shown as green spheres.

(D) Side view with the crystal structures of different Csn2 tetramers (Koo et al., 2012; PDB 3TOC and Ellinger et al., 2012; PDB 3QHQ) aligned to the top head domain of a Csn2 tetramer from the complexes described in this work. The concave side formed by different conformations of the Csn2A and Csn2B chains is conserved in all three structures but with varying degrees of rotation.

Figure S5. Related to Figure 1, Figure 2 and Figure 5

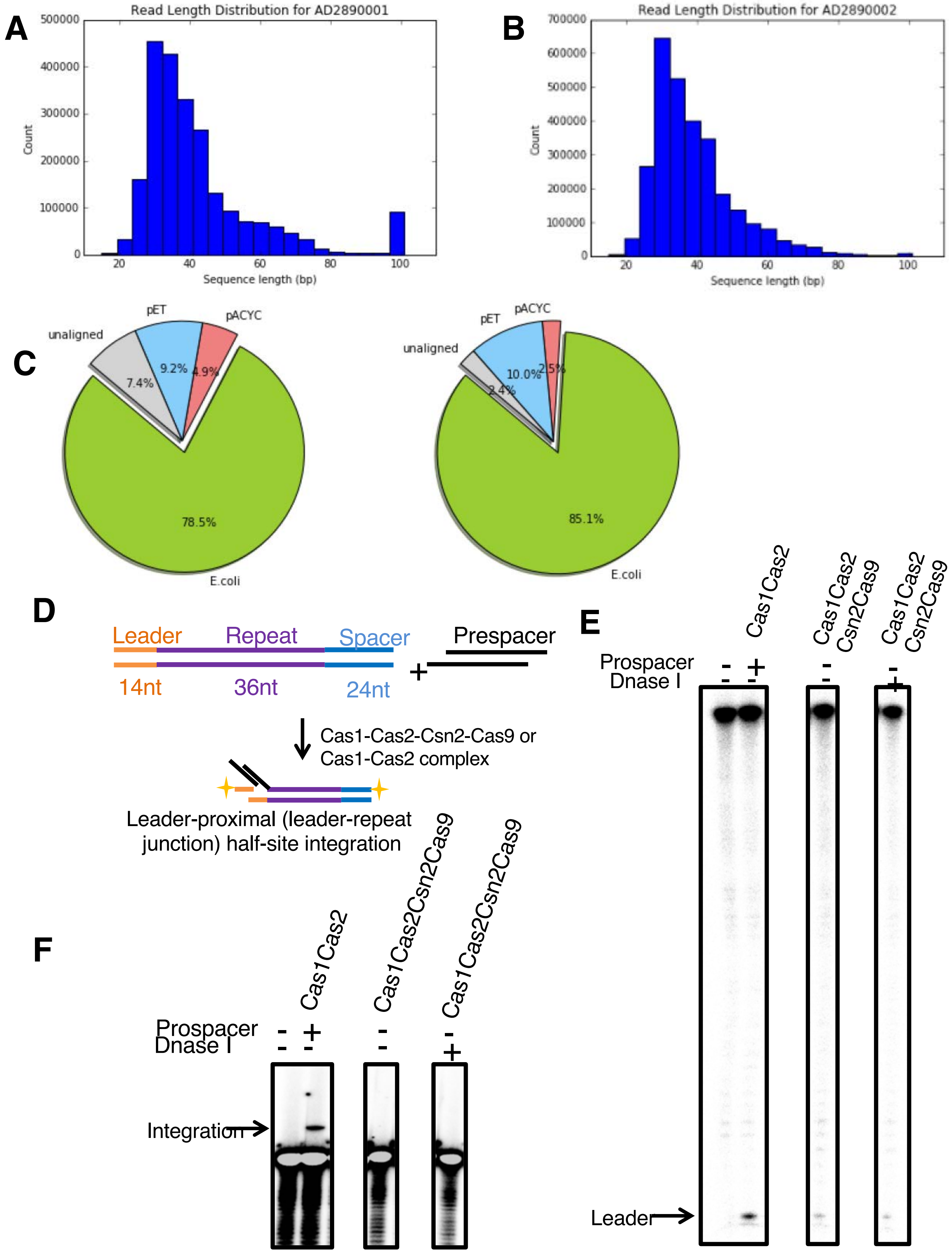

Figure S5. Deep-sequencing analysis of DNA captured by Cas1- Cas2-Csn2-Cas9 complex and integration assays. Related to Figure 1, Figure 2 and Figure 5.

(A) DNA captured by Cas1-Cas2-Csn2-Cas9 length distribution before DNAse I treatment. Many reads of 100 or more bp are evident. They are the likely filamentous structures seen in initial negative stain EM.

(B) DNA captured by Cas1-Cas2-Csn2-Cas9 length distribution after DNAse I treatment. The number of reads of 100+ length is decreased dramatically and the main distribution is more streamlined. This is consistent with the homogenization of DNA in the sample.

(C) Maps of the DNA captured by Cas1-Cas2-Csn2-Cas9 complexes onto *E. coli* genome and plasmids encoding CRISPR3-Cas system. As expected, DNA distribution closely matched expected DNA concentrations in the cell. No enrichment near PAM sequences was detected.

(D) Schematic representation of the integration assay. Prespacer was not added in case of Cas1-Cas2-Csn2-Cas9, as it was assumed that DNA bound by the complex may serve as one. 28

(E) Integration assay into substrates with labelled 5' end. Both complexes catalyse the nicking at the Leader-Repeat junction as evidenced by the release 14nt Leader fragment indicative of integration.

(F) Integration assay into substrates with labelled 3' ends. Only Cas1-Cas2 complex, but not the full complex exhibited permanent integrations resulting in the elongation of the initial substrate strand.

Figure S6. Related to Figure 1.

A

B

C

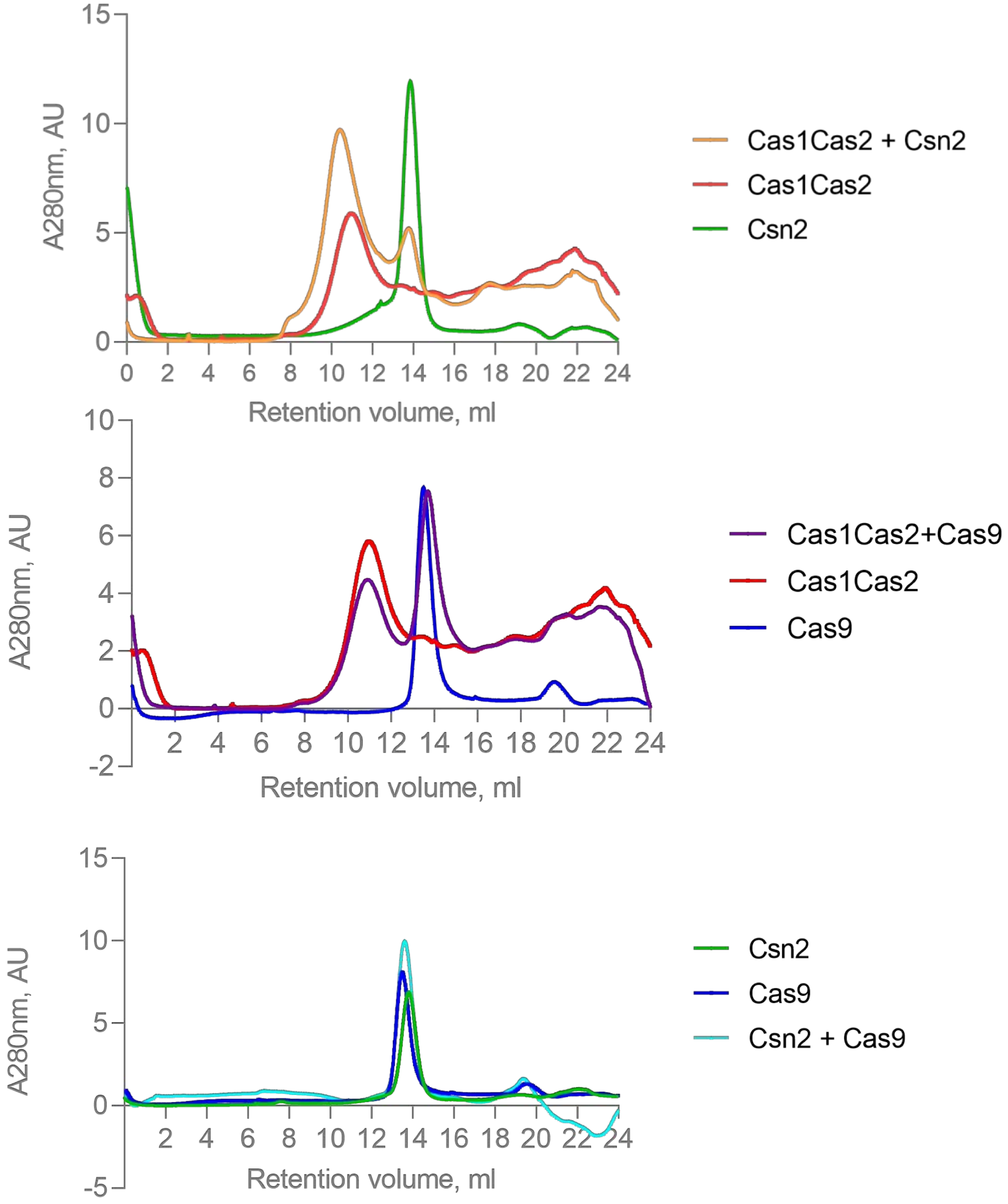

Figure S6. ***In vitro*** **interactions of CRISPR3-Cas proteins. Related to Figure 1.**
(A) Cas1-Cas2 interacts with Csn2 directly. Cas1-Cas2 eluted in the void volume, because they Had bound nucleic acids. However, upon mixing it with excess Csn2, a portion of Csn2 moved to the void volume as well.
(B) Cas1-Cas2 does not directly interact with Cas9. Cas9 peak neither decreased in size not moved to the left, therefore Cas9 did not interact with Cas1-Cas2.
(C) Csn2 does not interact with Cas9 directly, as the retention volume of the combined proteins did not shift left. Cas9 used for these experiments was complexed with tracr:crRNA duplex.

Figure S7. Related to Figure 5.

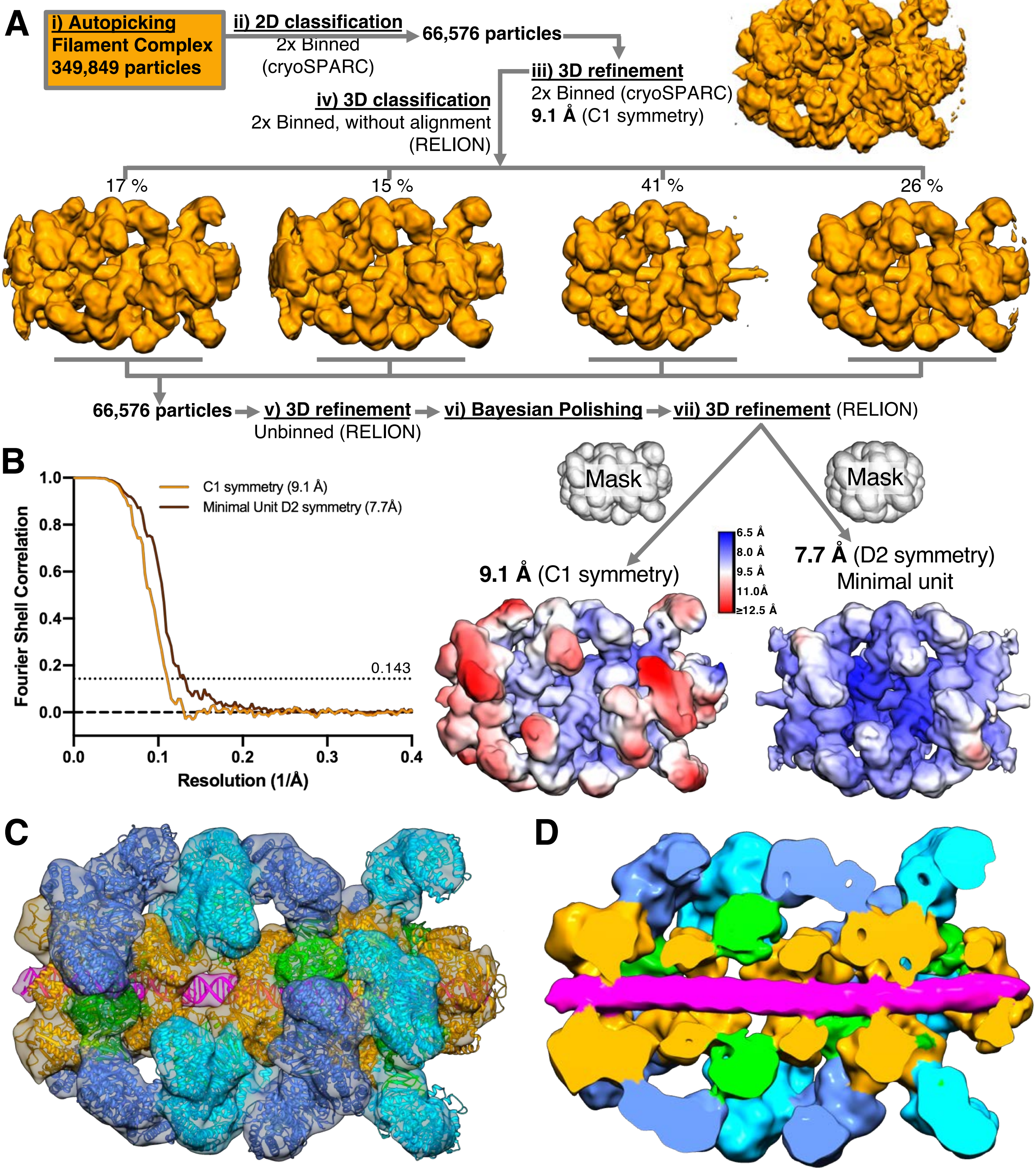

Figure S7. EM processing for the Cas1Cas2Csn2 filament complex. Related to Figure 5.

(A) Scheme overview of the initail processing steps for the filament particle set.

(B) FSC plot for the final filament maps (left). The deposited C1 and supplemental D2-symmetrised maps, coloured by local resolution (calculated using RELION), are displayed along with the corresponding masks used during refinement (right).

(C) The deposited 9.1 Å filament map is shown as a transparent surface with the coordinates underneath. Both are coloured similarly to Figure 5.

(D) Similar view to S7C of the deposited filament map, which is sliced in half to show the continuous density for dsDNA (magenta) through the entire filament.
