# Supplementary material for: Structure of the DNA-Bound Spacer Capture Complex of a Type II CRISPR-Cas System
Source: Mol Cell. 2019 Jul 11;75(1):90–101.e5. doi: 10.1016/j.molcel.2019.04.020 (PMC6620040; doi:10.1016/j.molcel.2019.04.020)
Supplement: Document S2. Article plus Supplemental Information [file mmc5.pdf]

Molecular Cell

Article

Structure of the DNA-Bound Spacer Capture Complex of a Type II CRISPR-Cas System

Graphical Abstract

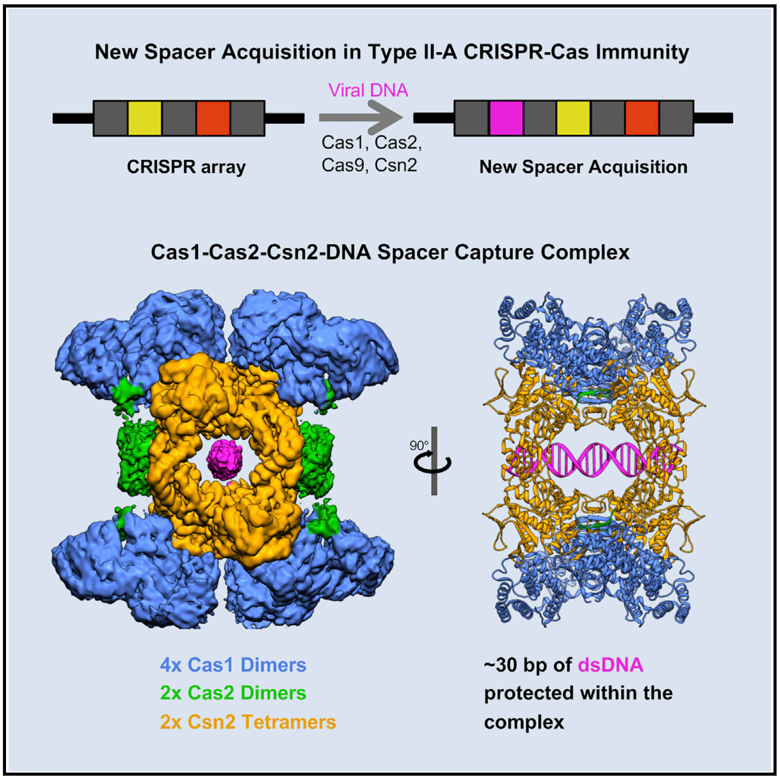

Authors

Martin Wilkinson,
Gediminas Drabavicius,
Arunas Silanskas, Giedrius Gasiunas,
Virginijus Siksnys, Dale B. Wigley

Correspondence

siksnys@ibt.lt (V.S.),
d.wigley@imperial.ac.uk (D.B.W.)

In Brief

The mechanism of spacer capture by type II CRISPR-Cas systems remains enigmatic. Wilkinson et al. report cryo-EM structures of three macromolecular assemblies of the Cas1, Cas2, and Csn2 proteins. All three complexes contain duplex DNA running along a central channel. A filamentous form contains a continuous duplex and smaller units contain shorter sections of ∼30 bp.

Highlights

- Cryo-EM structures of three different assemblies of the Cas1, Cas2, and Csn2 proteins
- One form, with the stoichiometry $Cas1_8$-$Cas2_4$-$Csn2_8$, contains ∼30 bp duplex DNA
- Another comprises two of these units with an additional $Cas1_8$-$Cas2_4$ interface
- Filaments comprise $Cas1_8$-$Cas2_4$-$Csn2_8$ units around a continuous DNA duplex

Wilkinson et al., 2019, Molecular Cell *75*, 90–101
July 11, 2019 © 2019 The Author(s). Published by Elsevier Inc.
https://doi.org/10.1016/j.molcel.2019.04.020

Molecular Cell
Article

Structure of the DNA-Bound Spacer Capture Complex of a Type II CRISPR-Cas System

Martin Wilkinson,[1] Gediminas Drabavicius,[2] Arunas Silanskas,[2] Giedrius Gasiunas,[2,3] Virginijus Siksnys,[2,*] and Dale B. Wigley[1,4,*]
[1]Section of Structural Biology, Department of Medicine, Imperial College London, London SW7 2AZ, UK
[2]Institute of Biotechnology, Vilnius University, Vilnius, Lithuania
[3]Present address: CasZyme, Vilnius, Lithuania
[4]Lead Contact
*Correspondence: siksnys@ibt.lt (V.S.), d.wigley@imperial.ac.uk (D.B.W.)
https://doi.org/10.1016/j.molcel.2019.04.020

SUMMARY

CRISPR and associated Cas proteins function as an adaptive immune system in prokaryotes to combat bacteriophage infection. During the immunization step, new spacers are acquired by the CRISPR machinery, but the molecular mechanism of spacer capture remains enigmatic. We show that the Cas9, Cas1, Cas2, and Csn2 proteins of a *Streptococcus thermophilus* type II-A CRISPR-Cas system form a complex and provide cryoelectron microscopy (cryo-EM) structures of three different assemblies. The predominant form, with the stoichiometry Cas1$_8$-Cas2$_4$-Csn2$_8$, referred to as monomer, contains ~30 bp duplex DNA bound along a central channel. A minor species, termed a dimer, comprises two monomers that sandwich a further eight Cas1 and four Cas2 subunits and contains two DNA ~30-bp duplexes within the channel. A filamentous form also comprises Cas1$_8$-Cas2$_4$-Csn2$_8$ units (typically 2–6) but with a different Cas1-Cas2 interface between them and a continuous DNA duplex running along a central channel.

INTRODUCTION

CRISPR-Cas is a set of microbial adaptive immune systems characterized by the insertion of short viral- or plasmid-derived DNA fragments called spacers into the CRISPR regions in the microbe's chromosome. These spacers serve as an immunological memory and protect the host via Cas proteins.

Fingerprinting invading DNA by inserting fragments as spacers into the CRISPR region is called adaptation (Barrangou et al., 2007). Spacers serve as templates for generation of CRISPR RNA (crRNA) guides for the interference machinery encoded by Cas proteins. On the basis of the composition of ribonucleoprotein complexes that constitute the interference machinery, CRISPR-Cas systems are divided (Makarova et al., 2015) into class 1 with multi-subunit interference complexes exemplified by Cascade (Brouns et al., 2008) and class 2 with single-protein multi-domain effectors, such as Cas9 (Gasiunas et al., 2012; Jinek et al., 2012) or Cas12 (Zetsche et al., 2015).

In a broad sense, the adaptation stage consists of the spacer capture step, where a prespacer is taken from the invading DNA, and the integration step, where a spacer is inserted into the CRISPR array. The prespacer acquired during the capture may require a separate step for end processing or may be processed directly in the integration complex (Drabavicius et al., 2018; Kieper et al., 2018; Lee et al., 2018; Rollie et al., 2018; Shiimori et al., 2018). Despite the differences between different CRISPR-Cas systems, spacer integration is likely to be achieved in relatively similar ways, due to the fact that it is carried out by the highly conserved Cas1 and Cas2 proteins (Krupovic et al., 2017; Yosef et al., 2012).

In a prototypical type I-E CRISPR-Cas system from *E. coli*, Cas1 and Cas2 form a complex that promotes spacer integration into the CRISPR array (Nuñez et al., 2015a; Yosef et al., 2012). In this complex, a Cas2 dimer is sandwiched by two Cas1 dimers in a butterfly-like arrangement to provide a platform for the binding of a prespacer in the form of a 23-bp duplex with 5-nt, splayed or 3′ protruding ends (Nuñez et al., 2015b; Wang et al., 2015). A prespacer contains a protospacer (a sequence matching the spacer in the CRISPR array) and a PAM (protospacer adjacent motif) sequence, the latter being needed for the discrimination between self and non-self (Nuñez et al., 2015b; Wang et al., 2015). One of the Cas1 subunits recognizes the PAM sequence, which is subsequently removed by an as yet unknown mechanism, and a protospacer sequence is integrated into the CRISPR array (Nuñez et al., 2015b; Wang et al., 2015). The protospacer integration reaction proceeds by nucleophilic attack of both sides of the first repeat of the CRISPR region by the free 3′-hydroxyl ends of the protospacer. This reaction is carried out by the Cas1-Cas2 complex, with Cas1 playing the catalytic role in the integration reaction. For the type I-E system, integration specificity is enhanced by the integration host factor (IHF) binding to the leader sequence of the CRISPR region to impose a specific DNA structure that is recognized by the Cas1-Cas2 complex (Nuñez et al., 2016). The resulting product is repaired by host factors, and each integration event is accompanied by the duplication of the leader-proximal repeat sequence (Amitai and Sorek, 2016; Sternberg et al., 2016).

The universal conservation of the Cas1 and Cas2 proteins implies that the spacer integration mechanism may share

 © 2019 The Author(s). Published by Elsevier Inc.
This is an open access article under the CC BY license (http://creativecommons.org/licenses/by/4.0/).

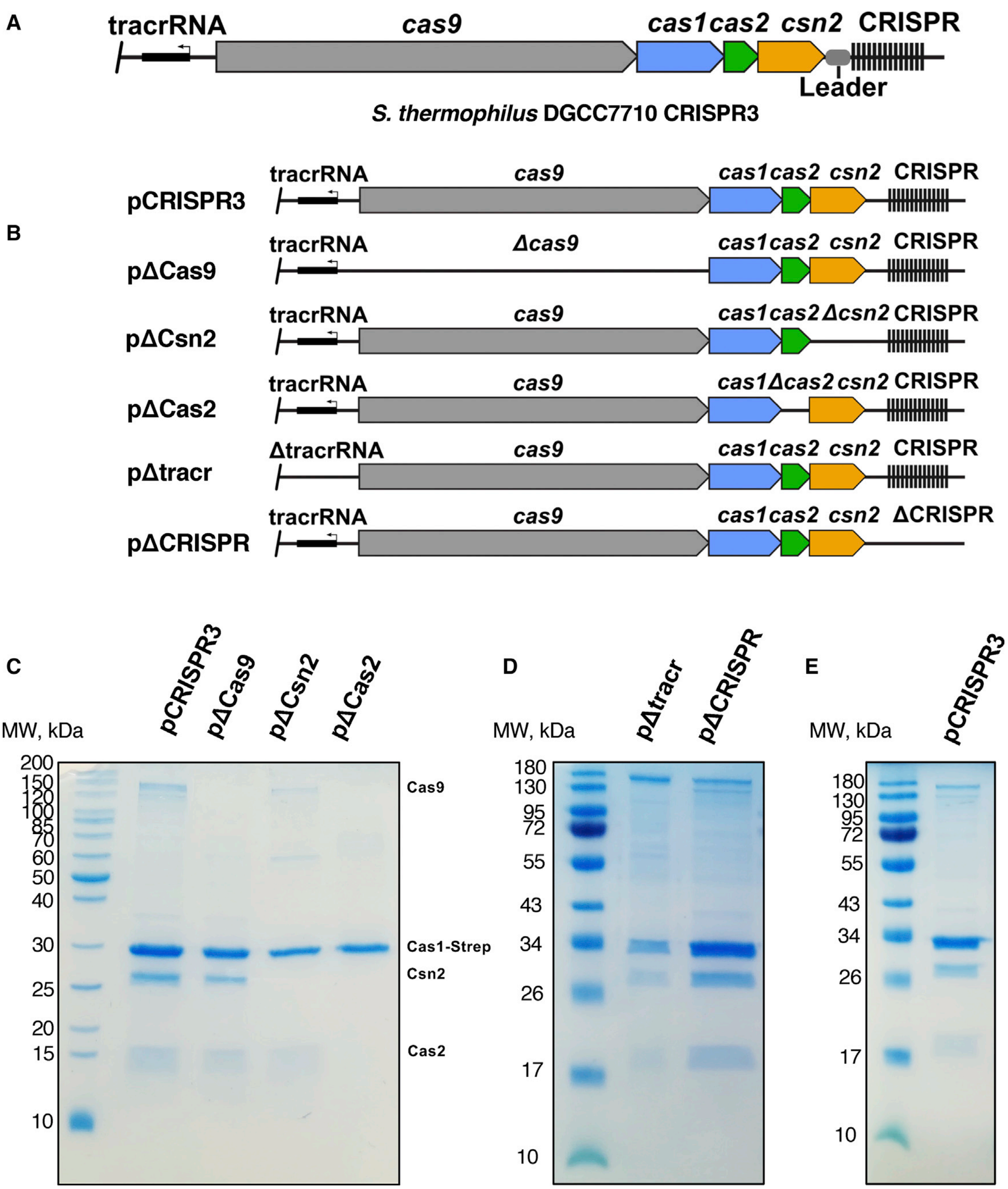

Figure 1. Cas1, Cas2, Csn2, and Cas9 Proteins of CRISPR3-Cas System from *S. thermophilus* DGCC7710 Form a Stable Complex

(A) Schematic representation of the CRISPR3-Cas locus.

(B) CRISPR3-Cas system deletion mutants used to screen the complex assembly.

(C) Results from purification of the complexes using the deletion mutants of the CRISPR3-Cas system depicted in (B). The CRISPR3-Cas system variants were co-expressed with Cas1 protein bearing a StrepII tag. The complexes were purified using StrepTrap column followed by size-exclusion chromatography and analyzed on SDS-PAGE. Cas1 and Cas2 form the core complex. Csn2 binds to Cas1-Cas2 complex (or vice versa), allowing binding of Cas9.

(D) Cas1, Cas2, Csn2, and Cas9 proteins co-purify in the absence of crRNA and tracrRNA.

(E) SDS-PAGE of the purified complex used for structural analysis.

similarities across different CRISPR-Cas systems. However, the type II-A CRISPR-Cas systems of *Streptococcus thermophilus* and *Streptococcus pyogenes* encode four Cas proteins: Cas9; Cas1; Cas2; and Csn2 (Figure 1A), all of which are essential for adaptation *in vivo* (Heler et al., 2015; Wei et al., 2015). However, although Cas9 nucleolytic activity is not required for protospacer integration, mutations of amino acid residues in Cas9, which are known to be involved in PAM recognition, lead

to PAM-independent spacer acquisition, confirming that Cas9 is required for insertion of spacers with a correct PAM sequence (Heler et al., 2015; Wei et al., 2015). Although Csn2 is indispensable in the adaptation step, its function remains obscure. Previous studies revealed that the Csn2 protein is tetrameric and forms a toroidal structure (Arslan et al., 2013; Ellinger et al., 2012; Lee et al., 2012; Nam et al., 2011). Although no biochemical activity has been detected for Csn2, it has been shown to bind to double-stranded DNA ends (Ellinger et al., 2012; Lee et al., 2012; Nam et al., 2011). Recent work has shown that all four Cas proteins in this system interact with one another (Ka et al., 2018).

The Cas1-Cas2 complex from the type II-A CRISPR-Cas system of *S. pyogenes* can integrate short DNA duplexes into the CRISPR region *in vitro*, similar to the *E. coli* Cas1-Cas2 complex. Importantly, the Cas9 or Csn2 proteins are not required for spacer integration *in vitro* (Wright and Doudna, 2016). The crystal structure of a Cas1-Cas2-prespacer complex from a type II-A CRISPR-Cas system from *Enterococcus faecalis* revealed an additional DNA binding interface that is employed in the recognition of the leader-repeat junction and the leader-distal part of the repeat, to ensure site-specific integration that is independent of host factors (Xiao et al., 2017). Taken together, these data show that the Cas1-Cas2 complex of type II-A CRISPR-Cas systems is sufficient for spacer integration into CRISPR arrays. However, the roles of the Cas9 and Csn2 proteins, both of which are required for spacer adaptation *in vivo*, remain to be established.

Here, we report the cryoelectron microscopy (cryo-EM) structure of the *Streptococcus thermophilus* Cas1-Cas2-Csn2 complex from the CRISPR3-Cas system. The predominant form of the complex is what we refer to as the "monomer" that contains the three proteins with the stoichiometry $Cas1_8$-$Cas2_4$-$Csn2_8$. Duplex DNA binds along a central channel formed within this complex that spans 25–30 bp. Minor species are also present. One form contains two of the monomer particles with an additional interface comprising a further four Cas1 dimers and two Cas2 dimers. This "dimer" complex ($Cas1_{24}$-$Cas2_{12}$-$Csn2_{16}$) contains two fragments of duplex DNA running through the center, and the additional pair of Cas2 dimers caps the ends of this DNA. Finally, a filamentous form comprises monomer units (typically 2–6) assembled around a continuous DNA duplex with an interface again comprising eight Cas1 and four Cas2 subunits but in a different configuration to the dimer complex. These structures provide insight into assembly of the spacer capture complex and the poorly understood process of spacer capture.

RESULTS

Analysis of Interactions between Cas9, Cas1, Cas2, and Csn2 Proteins in the *S. thermophilus* CRISPR3-Cas System

Although Cas9 and Csn2 proteins of type II-A systems are both required for the adaptation step *in vivo* (Heler et al., 2015; Wei et al., 2015), the Cas1-Cas2 complex alone is sufficient to integrate spacers *in vitro* (Wright and Doudna, 2016). To resolve this discrepancy, we investigated interactions between the four proteins by expressing a StrepII-tagged Cas1 protein in *E. coli* and then using this to pull down complexes from *E. coli*-expressing combinations of the other three proteins (Figure 1B). When all four proteins were co-expressed, a complex was isolated that contained all of them (Figure 1C). When we deleted Cas9, both Cas2 and Csn2 still co-purified with Cas1. However, in the absence of Csn2, mainly Cas1 and Cas2 co-purified accompanied by a marginal amount of Cas9 (Figure 1C). In the absence of Cas2, only Cas1 was recovered (Figure 1C). Furthermore, deletion of either CRISPR array or trans-activating crRNA (tracrRNA) did not impact complex formation, and we recovered all four proteins in the absence of either RNA (Figure 1D). These data show that Cas1 and Cas2 interact directly, and these proteins then interact with Csn2 followed by Cas9. This is further corroborated by analytic size-exclusion chromatography, whereupon Cas1-Cas2 complex binds Csn2, but not Cas9 (Figures S6A and S6B). This suggestion is in accord with the recent findings on a type II-A system from *S. pyogenes* showing a direct interaction between these proteins (Ka et al., 2018). However, in contrast, in the *S. thermophilus* CRISPR3-Cas system, we were unable to detect direct interaction between isolated Csn2 and Cas9 proteins (Figure S6).

Preparation of Cas1-Cas2-Csn2 Complex Sample for cryo-EM

To prepare the full complex for structural and functional analysis, the proteins were co-expressed along with a native CRISPR array containing twelve spacer-repeat units. SDS-PAGE analysis of the complex isolated from this expression system revealed all four proteins were present (Figure 1E).

Initial negative stain and cryo-EM analysis of this sample revealed heterogeneity in the size of particles and the presence of long threads that we presumed to be DNA (Figure S1A). These features precluded high-resolution analysis. Consequently, a clean-up step, involving DNase I treatment followed by gel filtration, was implemented prior to image analysis (Figure S1B). This treatment allowed the isolation of a homogeneous fraction eluting from gel filtration at the point expected for a complex of 400–500 kDa while removing a large mixture of digested DNA fragments, some subcomplexes, as well as higher molecular weight assemblies and/or aggregates. This sample was then used for the collection of a high-resolution cryo-EM dataset (see STAR Methods and Table 1). Processing of the images revealed that the majority of the particles related either to varying assemblies of Cas1-Cas2-Csn2 on double-stranded DNA (dsDNA) or free complexes of Cas9 with crRNA:tracrRNA duplex but with no complexes between the two (Figures S1C and S1D).

Architecture of the $Cas1_8$-$Cas2_4$-$Csn2_8$ Complex

The majority of the particles were of a complex with a stoichiometry of $Cas1_8$-$Cas2_4$-$Csn2_8$, and a 3.2-Å resolution density map was obtained from a homogeneous set of 306,470 of these particles after applying D2 symmetry (Figures S2 and S3). We refer to this as the "monomer" structure hereafter. The monomer complex forms an open, extended structure flanked on two sides by a Csn2 tetramer (Figures 2A and 2B; Video S1). The Cas1-Cas2 proteins assemble as essentially two dimers of $Cas1_4$-$Cas2_2$, as seen in previous structures (Nuñez et al., 2014), albeit with a different conformation (see below), that bridge the Csn2

Table 1. Cryo-EM Statistics for Data Collection and Model Refinement

	Cas1-Cas2-Csn2 Monomer (EMD-4668; PDB: 6QXF)	Cas1-Cas2-Csn2 Dimer (EMD-4670; PDB: 6QXT)	Cas1-Cas2-Csn2 Filament (EMD-4671; PDB: 6QY3)
Data Collection			
Microscope	Titan Krios	Titan Krios	Titan Krios
Camera	FEI Falcon III (integrating)	FEI Falcon III (integrating)	FEI Falcon III (integrating)
Voltage (kV)	300	300	300
Magnification	75,000	75,000	75,000
Pixel size (Å)	1.085	1.085	1.085
Electron dose (e^-/Å^2)	92.8	92.8	92.8
Dose rate (e^-/pixel/s)	109.2	109.2	109.2
Defocus range (μm)	−1.3 to −2.8	−1.3 to −2.8	−1.3 to −2.8
Final particles (no.)	306,389	3,109	66,576
Symmetry imposed	C1	C1	C1
Map resolution (Å)	3.6	8.9	9.1
Map sharpening B-factor (Å^2)	−80	−250	−300
Model Composition			
Nonhydrogen atoms	34,693	59,686	54,986
Protein residues	4,138	11,688[a]	10,664[a]
DNA bases	50	108	132
Ca^{2+} ligands	8	16	16
Refinement			
Initial PDB templates	PDB: 3V7F; PDB: 5XVN	PDB: 5ZYF; PDB: 6QXF	PDB: 6QXF
FSC (entire box)	0.90	0.85	0.80
FSC (around atoms)	0.87	0.71	0.66
RMS Deviations			
Bond lengths (Å)	0.002	0.001	0.001
Bond angles (°)	0.41	0.42	0.37
Validation			
Clashscore	6.9	0.9[a]	1.1[a]
Poor rotamers (%)	0.7	NA[a]	NA[a]
Ramachandran Plot			
Favored (%)	97.7	97.6	97.6
Allowed (%)	2.2	2.3	2.3
Disallowed (%)	0.1	0.1	0.1

FSC, Fourier shell correlation; RMS, root mean square
[a]Residues clipped at the C α positions

tetramers. The simple stoichiometry of the monomer is consistent with that proposed previously (Ka et al., 2018) but with a multiplicity of two. A channel runs through the complex that includes the central cavity within the Csn2 tetramers, and density for a DNA duplex is observed running along this channel (Figures 2 and S4). Each Csn2 tetramer is a dimer of dimers with two conformations ($Csn2_A$ and $Csn2_B$) that form two interwoven sets of head and leg domains (Figures 2C and S4), as described previously in crystal structures (Ellinger et al., 2012; Lee et al., 2012; Nam et al., 2011). The tetramers are also inverted with respect to one another so that each concave side faces inward, resulting in D2 symmetry of the protein components.

As suggested by atomic force microscopy (AFM) analysis of *S. agalactiae* Csn2 (Arslan et al., 2013), dsDNA threads through the central hole of successive Csn2 tetramers. This channel is 25–30 Å in diameter and ~90 Å in length that spans ~25 bp of DNA, although an additional couple of base pairs are observed protruding from each end of the channel. A surface charge representation shows a string of positive charges lining this channel (Figure S4). There appears to be only a limited number of protein contacts with the bound DNA, involving the conserved basic residues R55, K77, K131, and K160 of the Csn2 subunits (Figure S4). All the contacts appear to be electrostatic with the phosphate backbone of the DNA rather than sequence-specific DNA interactions. These characteristics are more akin to those of a DNA sliding clamp, such as PCNA (Moldovan et al., 2007), than to a specific DNA interaction. Although the majority of the particles contain bound duplex DNA, some particles are empty yet fully assembled (Figure S1E). This shows that assembly of the complex is not dependent upon DNA for stability, suggesting this

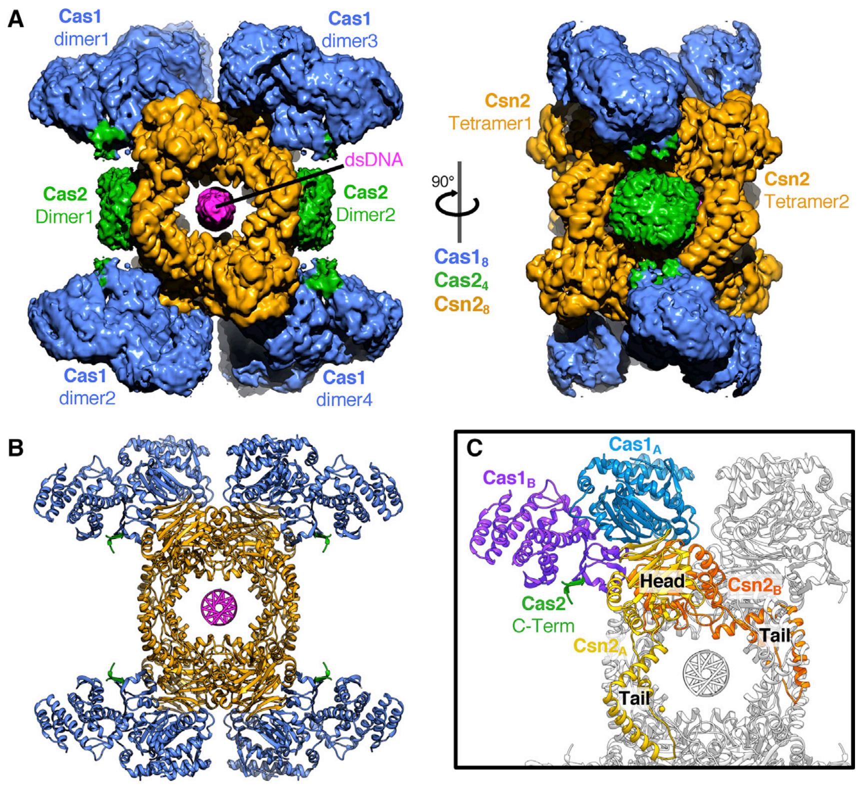

Figure 2. Structure of Cas1$_8$-Cas2$_4$-Csn2$_8$ Monomer

(A) Perpendicular views of the 3.2-Å-resolution cryo-EM map of the monomer complex. Density for the Cas2 dimers is shown but was not of sufficient quality for the subunits to be positioned uniquely.

(B) Cartoon of the final model colored as in (A). For Cas2, only the C-terminal Cas1-interacting region is shown.

(C) The 4-fold repeat unit of Cas1$_2$-Cas2$_1$-Csn2$_2$ is colored to highlight distinct conformations of the components.

might be the form that threads onto DNA ends rather than the complex assembling around DNA.

To determine whether there was any sequence preference for the bound DNA, we analyzed DNA sequences that co-purified with the complex by deep sequencing. The length distribution of sequencing reads closely matched results from cryo-EM, with free nucleic acid of lengths >100 bp almost completely gone after the DNase I treatment and with DNA fragments of predominantly 28–32 bp in length being significantly enriched (Figure S5). We mapped fragments onto the *E. coli* genome and plasmids and found that sources of the DNA inside the complex closely matched the likely relative DNA concentrations in the *E. coli* cells (Figure S5), but we were unable to identify any enrichment of sequences from the vicinity of PAM sequences. As a result, the DNA component has been modeled from a rigid body fit of idealized B-form linear dsDNA, but the sequence is one chosen at random so should not be inferred as representing that present in the structure. It is noteworthy that we prepared the samples recombinantly from *E. coli* and the purification included a sonication step that will have produced DNA fragments with ends suitable for loading of Cas1-Cas2-Csn2 complexes, and these non-specific DNA fragments may obscure any preference for PAM-derived sequences.

A Pair of Cas1-Cas2 Complexes Bridges the Csn2 Tetramers

The free Cas1-Cas2 complex comprises a Cas2 dimer flanked by a pair of Cas1 dimers (Nuñez et al., 2014). A pair of these Cas1$_4$-Cas2$_2$ complexes bridges the two Csn2 tetramers in the Cas1-Cas2-Csn2 complex via interactions between the Cas1 dimers and the head domains of each Csn2 tetramer (Figures 2C and 3A). However, the interaction is not symmetric, so each Cas1 subunit of the dimer interacts with a different surface of the Csn2 proteins (Figures 3B–3D). This asymmetry accommodates two different conformations for the Cas1 monomers in the dimer. The conformation of the dimer is most similar to that reported for *Ef*Cas1Cas2:DNA complexes (Xiao et al., 2017; Figure 3C), even though the Cas1 and Cas2 subunits are not contacting DNA in our structure. The complex is, therefore, stabilized by a series of interactions across multiple interfaces.

Csn2 Blocks Access of Cas1 and Cas2 to the Bound DNA

Despite being from different CRISPR-Cas systems, Cas2 in all structures to date is dimeric, and the dimer tethers two Cas1 dimers in an elongated butterfly-like shape (Nuñez et al., 2014, 2015b; Wang et al., 2015; Xiao et al., 2017; Figure 4A). In our type II-A structure, Cas2 plays a similar role within the full complex. However, the Cas2 dimer is rather mobile and density is weaker than for other components in the complex. This is not due to reduced occupancy of Cas2 because the C-terminal region that contacts the Cas1 subunits is well resolved with strong density at high resolution (Figure S2E). This interaction involves a β strand from the C terminus of Cas2 adding to a β sheet in the N-terminal domain of Cas1, as observed in structures of *Ef*Cas1-Cas2 (Xiao et al., 2017) that is conserved in our *S. thermophilus* structure (Figure 3C). The interaction interface between *S. pyogenes* Cas1 and Cas2 has been shown to be contained within the C terminus of Cas2 (residues 92–113; Ka et al., 2018). Unfortunately, the limited resolution of the map around Cas2 prevented unambiguous docking and/or modeling of the N-terminal domains of the dimers (Figures 4B and S2C). Nonetheless, the two blocks of density can each accommodate a Cas2 dimer, sitting opposite one another on the equatorial plane of the structure, held between two pairs of Cas1 dimers.

In the context of the full complex, Cas2 is positioned over a hole between the leg domains of adjacent Csn2 tetramers (Figure 4C). In this position, Cas2 blocks direct access to the central channel and the bound duplex DNA. The Cas2 dimers are 12 Å

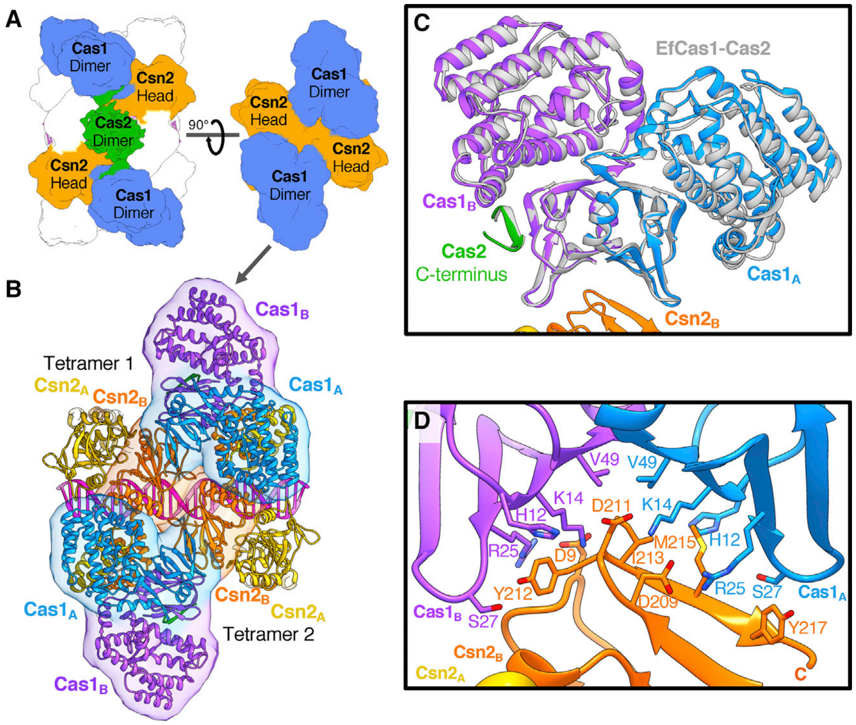

Figure 3. Interactions between Cas1-Cas2 and Csn2 Proteins within the Complex

(A) Cartoon showing the interactions between the subunits.

(B) More-detailed view of the top of the complex (A, right) showing how the Cas1 dimer interacts specifically with just the Csn2$_B$ protomer of each Csn2 head domain. The model is shown as a cartoon with the respective interacting chains outlined by a transparent molecular surface filtered to 20-Å resolution.

(C) An overlay of the EfCas1 dimer and Cas2 C terminus from the spacer-bound crystal structure (Xiao et al., 2017; PDB: 5XVN) with a Cas1 dimer from the monomer complex. Both the asymmetry of the Cas1 C-terminal domains (CTDs) and the interaction between the C-terminal strand of Cas2 and the Cas1$_B$ N-terminal domain are conserved.

(D) Close up of the Cas1-Csn2 interface. Different residues on a single Csn2 protein interact with the symmetrical surface of the Cas1 N-terminal domains (NTDs).

away from the DNA at the closest point, so Cas2 may play a gatekeeping role in this state of the complex, controlling or simply blocking access to the otherwise protected DNA duplex. In addition to being separated from Cas2, the DNA in the center of the complex is 90° rotated relative to the Cas1 active sites located on the outer edges of the complex (Figure 4B). It is therefore evident that this state does not reflect the active spacer integration states captured in other structures in the absence of Csn2 (Wright et al., 2017; Xiao et al., 2017).

This situation is indirectly supported by the fact that we have failed to detect direct spacer integration by the Cas1-Cas2-Csn2 complex using standard integration assays (Wright and Doudna, 2016; Xiao et al., 2017). To monitor spacer integration, we used a synthetic linear oligodeoxynucleotide substrate containing the last 14 nt of the leader sequence, a full repeat, and 24 nt of the first endogenous spacer (Figure S5D). If half-site integration occurred into the top strand at the leader-repeat junction, we would expect to observe a 14-nt fragment on a denaturing gel if the 5′ end were labeled and a >74-nt fragment if the 3′ end were labeled. Cas1-Cas2 subcomplex, but not Cas9-Cas1-Cas2-Csn2 complex, was able to integrate prespacers at the leader-repeat junction, resulting in either a 14-nt 5′-labeled fragment (Figure S5E) or elongation of the 3′-labeled strand (Figure S5F).

Cas1-Cas2-Csn2 Forms Larger Complex Assemblies on DNA

In addition to the monomer particles, we observed several other types of particles. Small particles were Cas9 protein (Figure S1D). In addition to the monomer complexes, a fraction of the Cas1-Cas2-Csn2 particles formed larger complexes: one formed discrete particles that were larger than the monomer complexes and the other was filamentous (Figure S1D). A structure for the larger discrete particles was obtained at 7.2-Å resolution (Figures 5 and S3; Video S2). These particles contain two sets of the Cas1$_8$-Cas2$_4$-Csn2$_8$ minimal complex bridged by a pair of Cas1$_4$-Cas2$_2$ subcomplexes, with the protein components again related by D2 symmetry (Figures 5A–5C). For simplicity, we refer to this particle hereafter as the “dimeric” state.

In the dimeric state, the additional Cas1 dimers dock at a site at the hinge between the Csn2 head and tail domains that is very different to the sites bound by the Cas1 subunits in the monomer (Figures 5 and 6). The same region of both Cas1 N-terminal domains (NTDs) in the dimer is located at the binding site but with a different site on Csn2. Together with the distinct Cas1-Csn2 interaction sites found in the monomer, this makes a total of four different interaction surfaces between Cas1 and Csn2. The alternate binding mode in the dimeric form does not induce any significant conformational changes within the monomer complexes, which, at this resolution, are unchanged relative to the free monomer. Duplex DNA is bound along a central channel but with two sections of DNA, each of ~30 bp, bound within the two monomers. Unlike the Cas2 dimers in the monomer, the additional pair of Cas2 dimers is not blocked from accessing the bound DNA by Csn2 and instead contacts the ends of the duplexes at the center of the dimer (Figures 5 and S3; Video S2).

Although the filaments were few in number, of variable in length, and less well ordered, some preliminary analysis was possible. A structure for the basic unit of the filaments, comprising two monomers and an interface between them, was obtained at 7.7-Å resolution. This filament repeat structure shows differences to the free dimers. First, the DNA running through the complex is a single continuous duplex that spans ~70 bp (Figures 5 and S7; Video S3). Second, the interface between monomers contains a pair of Cas1$_4$-Cas2$_2$ dimers but in a different configuration to the dimer. Attempts to morph between the two dimer

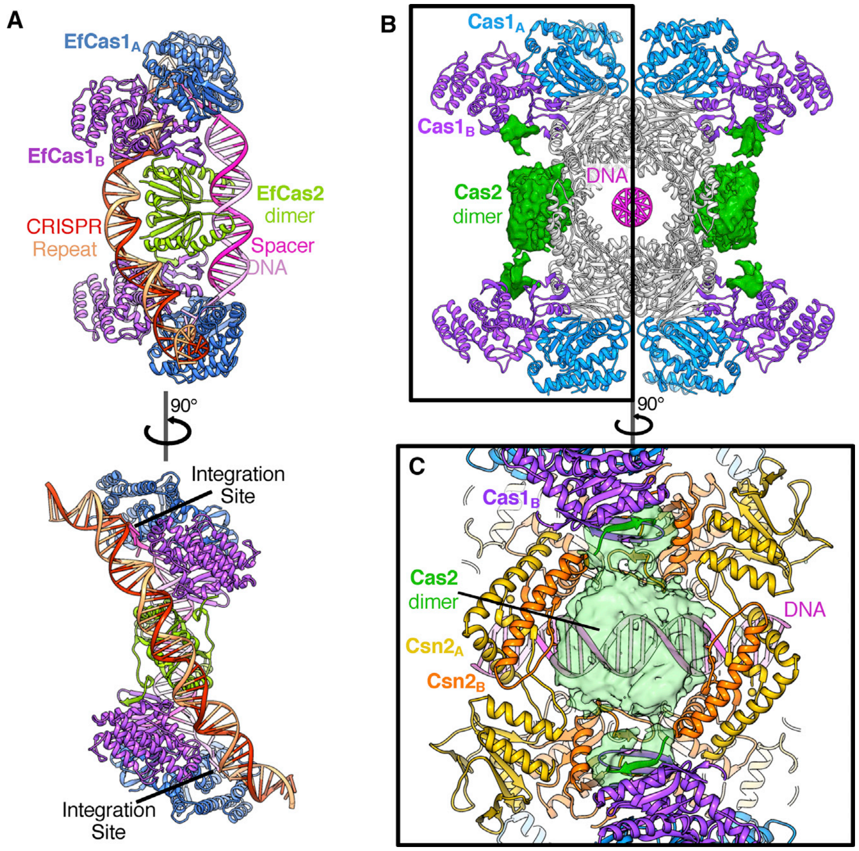

Figure 4. Conformational Analysis of the Cas1-Cas2 Proteins

(A) The structure of the EfCas1-Cas2 integration complex (Xiao et al., 2017; PDB: 5XVP).

(B) The Cas1-Cas2-Csn2 monomer model with Csn2 (gray) highlighting the positions of Cas1 and Cas2 relative to the bound DNA. The box denotes a single $Cas1_4$-$Cas2_2$ complex equivalent to the EfCas1-Cas2 structure in (A). The density corresponding to Cas2 sits between a pair of Cas1 dimers in a similar fashion to the EfCas1-Cas2 structures. However, the bound DNA is separate from Cas2 and rotated by 90° compared to the EfCas1-Cas2 integration structure.

(C) View from the side of the monomer complex with the Cas2 density shown as a transparent surface (green). Cas2 covers a pore between Csn2 tail domains of the adjacent tetramers, thereby blocking access to the bound DNA.

complexes, to model the conformational changes required to interchange between them, revealed that the changes required were too extensive to be a simple, one-step conversion between these forms. It is therefore unclear whether the filaments can transition in some way to release dimers or whether monomers are released that then reassemble into dimers.

DISCUSSION

A necessary but poorly understood stage prior to Cas1-Cas2-directed spacer integration is the acquisition (capture) of specific-length prespacer DNA substrates from the viral DNA adjacent to the PAM sites. In type I CRISPR systems, such as in *E. coli*, a complex of Cas1-Cas2 alone is sufficient to integrate new spacers into the CRISPR array (Nuñez et al., 2015a; Yosef et al., 2012). By contrast, in type II-A CRISPR systems, Cas1 proteins are unable to recognize the PAM sequence and so depend on the PAM-recognition function of Cas9 to generate new spacers (Heler et al., 2015; Wei et al., 2015). Surprisingly, spacer acquisition also requires the tetrameric dsDNA-binding protein Csn2 (Wei et al., 2015), whose role in the process is less clear. It has been shown that, once a correctly processed protospacer is generated, the Cas1-Cas2 subcomplex alone is sufficient to carry out the integration stage into the host CRISPR array (Wright and Doudna, 2016).

Although it has been shown that Cas1, Cas2, Csn2, and Cas9 are all required for spacer acquisition in type II-A CRISPR-Cas systems (Heler et al., 2015; Wei et al., 2015), the details of how prespacers are produced from viral DNA by these proteins remains unclear. Our biochemical analysis of the complex in cell extracts shows that the Cas1, Cas2, Csn2, and Cas9 proteins interact and co-purify with one another (Figures 1C and 1D). The $Cas1_8$-$Cas2_4$-$Csn2_8$ (monomer) complex structure we present here shows how these three proteins interact to form a large multi-subunit complex. Furthermore, the presence of double-stranded DNA running along the central channel of the complex suggests a protective role for the complex. The length of occluded DNA is approximately 30 bp, very similar to the length of the prespacer fragments produced as substrates for integration by Cas1-Cas2 complex (Wright and Doudna, 2016). Although, at this stage, several questions remain regarding how these fragments are produced, one function for the complex is evident. Bacteria contain RecBCD and/or AddAB nuclease-helicase complexes whose role, in addition to DNA repair, is digestion of invading bacteriophage DNA (Dillingham and Kowalczykowski, 2008). These enzyme complexes are highly effective DNA-degrading machines, as evidenced by the fact that several bacteriophages produce specific protein inhibitors of RecBCD and/or AddAB to prevent degradation of their genomes after infection (Poteete et al., 1988). The formation of free prespacers would be susceptible to degradation by RecBCD and/or AddAB, so a protective role for the $Cas1_8$-$Cas2_4$-$Csn2_8$ complex we present here could be beneficial. Interestingly, RecBCD has already been implicated as providing short spacers for integration into CRISPR arrays in *E. coli*, although the mechanism of this process remains unclear (Levy et al., 2015) and *E. coli* does not encode Csn2 within its CRISPR-Cas systems. Thus, it seems likely that there is a balance between the degradation of viral DNA by RecBCD and/or AddAB and requirements for spacer capture, which may relate to the differences in PAM recognition and prespacer processing observed for different systems.

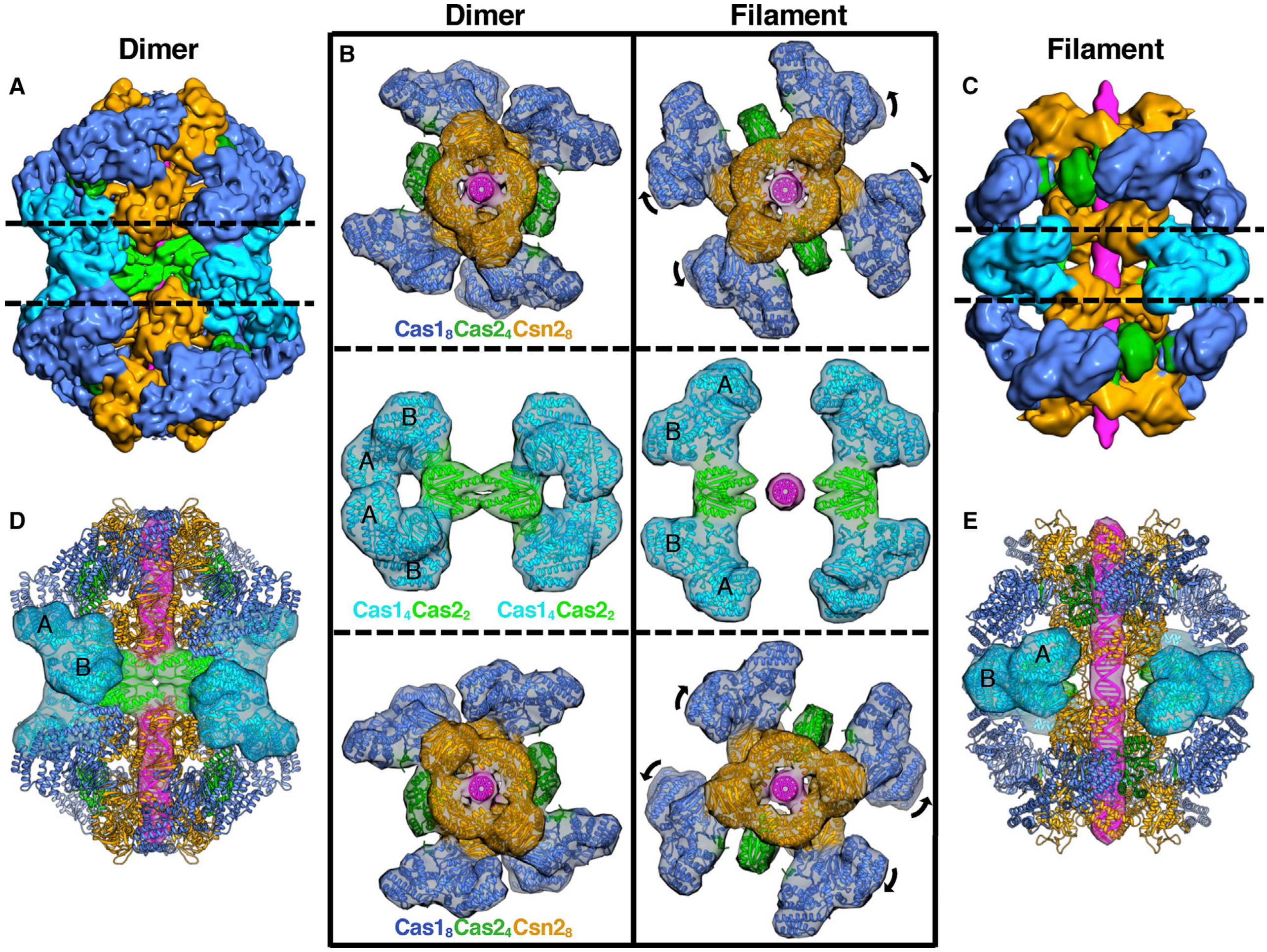

Figure 5. Comparison of the Dimer and Filament Complex Assemblies

(A) The 7.2-Å resolution dimer cryo-EM map colored by subunit.

(B) The dimer and filament complexes are split into three sections and rotated 90° relative to (A) and (C). Both assemblies share similar components but with different conformational arrangements. In the filament form, the monomer Cas1-Cas2-Csn2 unit shows a further rotation of the Csn2 tetramers relative to each other compared to both the dimer and monomer forms.

(C) The 7.7-Å resolution minimal unit masked filament cryo-EM map colored by subunit with a similar view to that of the dimer complex shown in (A).

(D) The dimer model is shown colored by subunit with transparent molecular surfaces calculated at 18-Å resolution shown to highlight the middle pair of Cas1-Cas2 subcomplexes. The Cas2 dimers cap the ends of two bound DNA fragments.

(E) The minimal filament model is shown colored by subunit with transparent molecular surfaces calculated at 18-Å resolution shown to highlight the middle pair of Cas1-Cas2 subcomplexes.

When presented with correctly processed spacers, Cas1-Cas2 complexes alone can successfully integrate substrates into CRISPR arrays (Nuñez et al., 2014, 2015b; Wang et al., 2015; Wright and Doudna, 2016), and several structures relating to the various intermediate stages have been determined (Wright et al., 2017; Xiao et al., 2017). Csn2 has a marked preference for DNA ends (Arslan et al., 2013; Koo et al., 2012; Nam et al., 2011), suggesting an active, if not catalytic, role in the stage of capturing spacers. Recent studies of a type II-A CRISPR-Cas system from *S. pyogenes* has suggested that Csn2 inhibits the integration reaction by Cas1-Cas2 (Wright and Doudna, 2016). Taken together, these data suggest that Csn2 pre-binding to the DNA ends eliminates Cas1-Cas2 binding to the prespacer, so our $Cas1_8$-$Cas2_4$-$Csn2_8$ complex may represent an early stage of spacer capture, providing a link between the potential spacer capture and integration complexes involving just Cas1-Cas2. If, in our structure, the flanking Csn2 tetramers were to dissociate and slide along the DNA, the Cas1-Cas2 units would be free to swing down to interact with the DNA to form the spacer substrate bound state. The Cas2 subunits are located appropriately to direct this process by engaging the DNA directly and then allowing the Cas1 dimers to engage the DNA substrate once the Csn2 tetramers have left.

The larger Cas1-Cas2-Csn2 "dimer" assembly shows that additional Cas1-Cas2 subcomplexes can dock between a pair of the $Cas1_8$-$Cas2_4$-$Csn2_8$ complexes and are able to access the ends of the centrally bound DNA via the Cas2 dimers (Figures 5 and S3; Video S2). The first structure of a Cas2 protein

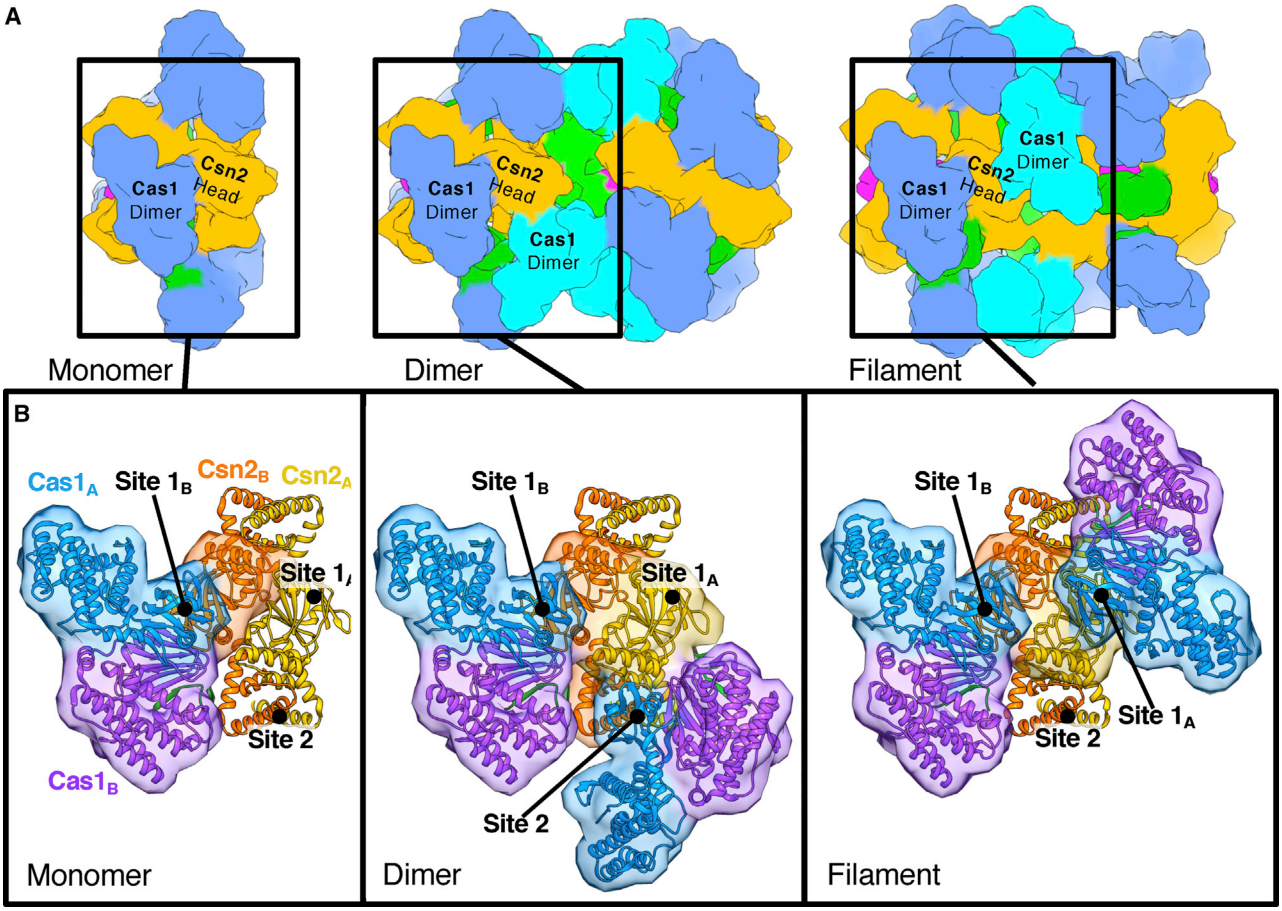

Figure 6. The Different Cas1 Docking Sites Dictate the Different Cas1-Cas2-Csn2 Assemblies

(A) Molecular surfaces calculated at 15 Å of the three Cas1-Cas2-Csn2 complexes colored by subunit as in previous figures. One Csn2 head domain with interacting Cas1 dimers is highlighted.

(B) Close-up view of the models with just the labeled regions in (A) shown for clarity and colored by protein. The transparent molecular surface calculated at 15-Å resolution is shown only for interacting regions. The three observed binding sites are labeled in each case.

from *Sulfolobus solfataricus* revealed a ferredoxin-like fold with RNase activity preferentially for single-stranded RNA (ssRNA) (Beloglazova et al., 2008). Numerous Cas2 structures have followed from different CRISPR systems (Jung et al., 2016; Ka et al., 2014, 2017; Samai et al., 2010), and the potential nucleolytic activity has been reported with differing specificities (Babu et al., 2011). In one SpCas2 structure, where ssRNase activity was reported (Ka et al., 2014), the conserved catalytic aspartate residues are 6.5 Å apart, leading to the proposal of one active site per Cas2 dimer with potential Mg^{2+} and water coordination to instigate a nucleophilic attack for scissile bond cleavage. More recently, the structure of a dimer of the N-terminal domains of another SpCas2 shows even closer approach of these acidic residues (3.6 Å; Ka et al., 2018). The conformations in other Cas2 structures from different organisms show separations greater than 10 Å and without conclusive demonstration of nucleolytic activity. Interestingly, these aspartate residues in the Cas2 subunits are shown to coordinate magnesium ions to the phosphodiester backbone of the DNA in the Cas1-Cas2 integration complex (even though no Cas2 nuclease activity is required for integration), and a function for these bound magnesium ions remains unclear (Xiao et al., 2017). Despite the medium resolution of the dimeric Cas1-Cas2-Csn2 structure, it is clear that the conformation of the middle Cas2 dimers in our structure is more similar to that in SpCas2 (Ka et al., 2018) but differs to that in other Cas2 structures and in the Cas1-Cas2 integration complexes. Such a conformational difference might relate to some form of regulation of nucleolytic activity because it is unlikely that both conformations would show the same activity on nucleic acid substrates, given the differences in metal ion separation distances.

The Cas9, Cas1, Cas2, and Csn2 proteins of type II-A systems are all required for the adaptation step *in vivo* (Heler et al., 2015; Wei et al., 2015), implying possible interactions between these proteins. Indeed, recent *in vitro* studies revealed direct interactions between these proteins in type II-A system from *S. pyogenes* (Ka et al., 2018). Here, using a StrepII-tagged version of the Cas1 protein, we pulled down the Cas9-Cas1-Cas2-Csn2 complex of type II-A system from *S. thermophilus* when expressed in the heterologous host. We aimed to solve

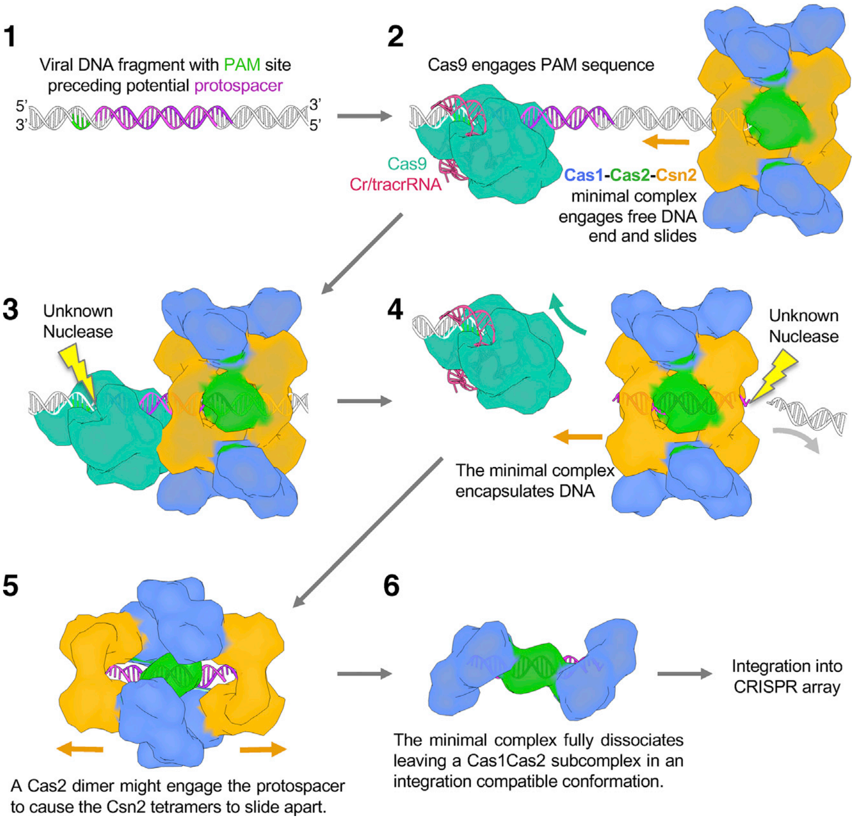

Figure 7. Proposed Scheme for Spacer Acquisition

A suggested scheme for the acquisition of spacers based on the monomer Cas1-Cas2-Csn2 structure.

the cryo-EM structure of this complex and to reduce sample heterogenicity by treatment with DNase I. In the resulting structures, the Cas9 protein (that was part of the pull-down complex) is missing. The cryo-EM structure of the major species revealed an unexpected arrangement of the Cas1, Cas2, and Csn2 proteins, forming a complex with a channel that is occupied by ~30-bp DNA duplex. However, we were also able to identify particles that contained stand-alone Cas9 protein bound to DNA. We assume that, in the Cas9-Cas1-Cas2-Csn2 complex that we pulled down, Cas9 was tethered to the complex via a DNA molecule and subsequent DNase treatment broke the tether to release the Cas9-DNA and Cas1-Cas2-Csn2 subcomplexes. In the major Cas1-Cas2-Csn2 subcomplex, two $Cas1_2$-$Cas2_4$ dimers encircle DNA and are capped by the Csn2 tetramers on each end. Cas9 is not required for an assembly of such a complex because we were able to isolate the Cas1-Cas2-Csn2 subcomplex from the host lacking the *cas9* gene. Given the Cas1-Cas2-Csn2 complex structure and the affinity of Csn2 for DNA ends (Ellinger et al., 2012; Lee et al., 2012; Nam et al., 2011), we propose a speculative model for spacer capture (Figure 7). Due to the high DNA end affinity of the Csn2 protein, the Cas1-Cas2-Csn2 complex engages free DNA ends in the cell and encircles DNA within the complex. An alternative model in which Csn2 could bind DNA ends first followed by assembly of the Cas1-Cas2-Csn2 components around this complex can probably be excluded because we observe some fully formed Cas1-Cas2-Csn2 complexes that lack DNA (see above and Figure S1E). The Cas1-Cas2-Csn2 complex could then slide on the DNA until it encounters Cas9 protein that is bound to a PAM sequence. At that point, the DNA could be cleaved (by an as yet unidentified cellular nuclease), releasing Cas9 and the Cas1-Cas2-Csn2 complex, encapsulating a ~30-bp DNA fragment as a new spacer ready for integration. Integration of the spacer would require dissociation of the complex to release the Cas1-Cas2 complex in a conformation compatible with spacer integration.

Taken together, our cryo-EM structures reveal an unexpectedly complex arrangement of the Cas1, Cas2, and Csn2 proteins of a type II-A CRISPR-Cas system and suggest a role for this complex in either spacer capture, processing, and/or acquisition that is much more complicated than current understanding suggests from previous studies of much smaller stand-alone Cas1-Cas2 complexes. These structures imply that, although the spacer integration step is conserved across the different CRISPR-Cas types and requires a conserved Cas1-Cas2 complex, spacer adaptation complexes may have quite different architectures. Recent studies of Cas4-dependent type I adaption complexes imply a complex variant that involves Cas4 protein together with a Cas1-Cas2 protein complex (Lee et al., 2018; Shiimori et al., 2018). The structures we present here shed light on the initial stages of spacer capture and reveal an unprecedented higher order assembly of Cas1-Cas2-Csn2 proteins that appears to play a role in this process. Nonetheless, it is also clear that further work will be required to elucidate details of how DNA captured in these structures becomes incorporated into CRISPR arrays.

STAR★METHODS

Detailed methods are provided in the online version of this paper and include the following:

- KEY RESOURCES TABLE
- CONTACT FOR REAGENT AND RESOURCE SHARING
- METHOD DETAILS
  - Isolation of Cas9-Cas1-Cas2-Csn2 and its derivative complexes
  - DNase I treatment prior to electron microscopy
  - Cryo-electron microscopy grid preparation and data collection
  - Data processing – Cas1-Cas2-Csn2-DNA monomer complex
  - Model Building – Cas1-Cas2-Csn2-DNA monomer complex

- ○ Data processing – Cas1-Cas2-Csn2-DNA dimer complex
- ○ Model Building – Cas1-Cas2-Csn2-DNA dimer complex
- ○ Data processing – Cas1-Cas2-Csn2-DNA filament complexes
- ○ Model Building – Cas1-Cas2-Csn2-DNA filament complexes
- ○ Sample library preparation for deep sequencing analysis
- ○ Integration reactions into linear substrates
- DATA AND SOFTWARE AVAILABILITY

SUPPLEMENTAL INFORMATION

Supplemental Information can be found online at https://doi.org/10.1016/j.molcel.2019.04.020.

ACKNOWLEDGMENTS

We thank Diamond for access and support of the Cryo-EM facilities at the UK national electron bio-imaging centre (eBIC), funded by the Wellcome Trust, MRC, and BBSRC, Rafael Ayala for assistance with data collection, and Shane Dooley (Iowa State University) for the NGS sequencing. The work was funded by the Medical Research Council (MR/N009258/1 to D.B.W.).

AUTHOR CONTRIBUTIONS

M.W. conducted the electron microscopy and data processing. G.G. and A.S. performed purification and initial characterization of the CRISPR3-Cas complex. G.D. performed biochemical assays and DNA sequencing and analyzed the results. M.W., G.D., G.G., V.S., and D.B.W. analyzed the data and wrote the manuscript. All authors read and approved the manuscript.

DECLARATION OF INTERESTS

G.G. and V.S. are inventors on patent applications related to CRISPR and co-founders of CasZyme. G.G. is an employee of CasZyme.

Received: November 9, 2018
Revised: March 8, 2019
Accepted: April 11, 2019
Published: May 9, 2019

REFERENCES

Afonine, P.V., Poon, B.K., Read, R.J., Sobolev, O.V., Terwilliger, T.C., Urzhumtsev, A., and Adams, P.D. (2018). Real-space refinement in PHENIX for cryo-EM and crystallography. Acta Crystallogr. D Struct. Biol. *74*, 531–544.

Amitai, G., and Sorek, R. (2016). CRISPR-Cas adaptation: insights into the mechanism of action. Nat. Rev. Microbiol. *14*, 67–76.

Arslan, Z., Wurm, R., Brener, O., Ellinger, P., Nagel-Steger, L., Oesterhelt, F., Schmitt, L., Willbold, D., Wagner, R., Gohlke, H., et al. (2013). Double-strand DNA end-binding and sliding of the toroidal CRISPR-associated protein Csn2. Nucleic Acids Res. *41*, 6347–6359.

Babu, M., Beloglazova, N., Flick, R., Graham, C., Skarina, T., Nocek, B., Gagarinova, A., Pogoutse, O., Brown, G., Binkowski, A., et al. (2011). A dual function of the CRISPR-Cas system in bacterial antivirus immunity and DNA repair. Mol. Microbiol. *79*, 484–502.

Barrangou, R., Fremaux, C., Deveau, H., Richards, M., Boyaval, P., Moineau, S., Romero, D.A., and Horvath, P. (2007). CRISPR provides acquired resistance against viruses in prokaryotes. Science *315*, 1709–1712.

Beloglazova, N., Brown, G., Zimmerman, M.D., Proudfoot, M., Makarova, K.S., Kudritska, M., Kochinyan, S., Wang, S., Chruszcz, M., Minor, W., et al. (2008). A novel family of sequence-specific endoribonucleases associated with the clustered regularly interspaced short palindromic repeats. J. Biol. Chem. *283*, 20361–20371.

Brouns, S.J.J., Jore, M.M., Lundgren, M., Westra, E.R., Slijkhuis, R.J.H., Snijders, A.P.L., Dickman, M.J., Makarova, K.S., Koonin, E.V., and van der Oost, J. (2008). Small CRISPR RNAs guide antiviral defense in prokaryotes. Science *321*, 960–964.

Dillingham, M.S., and Kowalczykowski, S.C. (2008). RecBCD enzyme and the repair of double-stranded DNA breaks. Microbiol. Mol. Biol. Rev. *72*, 642–671.

Drabavicius, G., Sinkunas, T., Silanskas, A., Gasiunas, G., Venclovas, Č., and Siksnys, V. (2018). DnaQ exonuclease-like domain of Cas2 promotes spacer integration in a type I-E CRISPR-Cas system. EMBO Rep. *19*, e45543.

Ellinger, P., Arslan, Z., Wurm, R., Tschapek, B., MacKenzie, C., Pfeffer, K., Panjikar, S., Wagner, R., Schmitt, L., Gohlke, H., et al. (2012). The crystal structure of the CRISPR-associated protein Csn2 from Streptococcus agalactiae. J. Struct. Biol. *178*, 350–362.

Emsley, P., Lohkamp, B., Scott, W.G., and Cowtan, K. (2010). Features and development of Coot. Acta Crystallogr. D Biol. Crystallogr. *66*, 486–501.

Gasiunas, G., Barrangou, R., Horvath, P., and Siksnys, V. (2012). Cas9-crRNA ribonucleoprotein complex mediates specific DNA cleavage for adaptive immunity in bacteria. Proc. Natl. Acad. Sci. USA *109*, E2579–E2586.

Goddard, T.D., Huang, C.C., and Ferrin, T.E. (2007). Visualizing density maps with UCSF Chimera. J. Struct. Biol. *157*, 281–287.

Heler, R., Samai, P., Modell, J.W., Weiner, C., Goldberg, G.W., Bikard, D., and Marraffini, L.A. (2015). Cas9 specifies functional viral targets during CRISPR-Cas adaptation. Nature *519*, 199–202.

Jinek, M., Chylinski, K., Fonfara, I., Hauer, M., Doudna, J.A., and Charpentier, E. (2012). A programmable dual-RNA-guided DNA endonuclease in adaptive bacterial immunity. Science *337*, 816–821.

Jung, T.-Y., Park, K.-H., An, Y., Schulga, A., Deyev, S., Jung, J.-H., and Woo, E.-J. (2016). Structural features of Cas2 from Thermococcus onnurineus in CRISPR-cas system type IV. Protein Sci. *25*, 1890–1897.

Ka, D., Kim, D., Baek, G., and Bae, E. (2014). Structural and functional characterization of Streptococcus pyogenes Cas2 protein under different pH conditions. Biochem. Biophys. Res. Commun. *451*, 152–157.

Ka, D., Hong, S., Jeong, U., Jeong, M., Suh, N., Suh, J.-Y., and Bae, E. (2017). Structural and dynamic insights into the role of conformational switching in the nuclease activity of the *Xanthomonas albilineans* Cas2 in CRISPR-mediated adaptive immunity. Struct. Dyn. *4*, 054701.

Ka, D., Jang, D.M., Han, B.W., and Bae, E. (2018). Molecular organization of the type II-A CRISPR adaptation module and its interaction with Cas9 via Csn2. Nucleic Acids Res. *46*, 9805–9815.

Karvelis, T., Gasiunas, G., Miksys, A., Barrangou, R., Horvath, P., and Siksnys, V. (2013). crRNA and tracrRNA guide Cas9-mediated DNA interference in *Streptococcus thermophilus*. RNA Biol. *10*, 841–851.

Kelley, L.A., Mezulis, S., Yates, C.M., Wass, M.N., and Sternberg, M.J.E. (2015). The Phyre2 web portal for protein modeling, prediction and analysis. Nat. Protoc. *10*, 845–858.

Kieper, S.N., Almendros, C., Behler, J., McKenzie, R.E., Nobrega, F.L., Haagsma, A.C., Vink, J.N.A., Hess, W.R., and Brouns, S.J.J. (2018). Cas4 facilitates PAM-compatible spacer selection during CRISPR adaptation. Cell Rep. *22*, 3377–3384.

Koo, Y., Jung, D.-K., and Bae, E. (2012). Crystal structure of Streptococcus pyogenes Csn2 reveals calcium-dependent conformational changes in its tertiary and quaternary structure. PLoS ONE *7*, e33401.

Krupovic, M., Béguin, P., and Koonin, E.V. (2017). Casposons: mobile genetic elements that gave rise to the CRISPR-Cas adaptation machinery. Curr. Opin. Microbiol. *38*, 36–43.

Lee, K.-H., Lee, S.-G., Eun Lee, K., Jeon, H., Robinson, H., and Oh, B.-H. (2012). Identification, structural, and biochemical characterization of a group of large Csn2 proteins involved in CRISPR-mediated bacterial immunity. Proteins *80*, 2573–2582.

Lee, H., Zhou, Y., Taylor, D.W., and Sashital, D.G. (2018). Cas4-dependent prespacer processing ensures high-fidelity programming of CRISPR arrays. Mol. Cell *70*, 48–59.e5.

Levy, A., Goren, M.G., Yosef, I., Auster, O., Manor, M., Amitai, G., Edgar, R., Qimron, U., and Sorek, R. (2015). CRISPR adaptation biases explain preference for acquisition of foreign DNA. Nature *520*, 505–510.

Li, X., Mooney, P., Zheng, S., Booth, C.R., Braunfeld, M.B., Gubbens, S., Agard, D.A., and Cheng, Y. (2013). Electron counting and beam-induced motion correction enable near-atomic-resolution single-particle cryo-EM. Nat. Methods *10*, 584–590.

Makarova, K.S., Wolf, Y.I., Alkhnbashi, O.S., Costa, F., Shah, S.A., Saunders, S.J., Barrangou, R., Brouns, S.J., Charpentier, E., Haft, D.H., et al. (2015). An updated evolutionary classification of CRISPR-Cas systems. Nat. Rev. Microbiol. *13*, 722–736.

Moldovan, G.L., Pfander, B., and Jentsch, S. (2007). PCNA, the maestro of the replication fork. Cell *129*, 665–679.

Murshudov, G.N., Vagin, A.A., and Dodson, E.J. (1997). Refinement of macromolecular structures by the maximum-likelihood method. Acta Crystallogr. D Biol. Crystallogr. *53*, 240–255.

Nam, K.H., Kurinov, I., and Ke, A. (2011). Crystal structure of clustered regularly interspaced short palindromic repeats (CRISPR)-associated Csn2 protein revealed Ca2+-dependent double-stranded DNA binding activity. J. Biol. Chem. *286*, 30759–30768.

Nuñez, J.K., Kranzusch, P.J., Noeske, J., Wright, A.V., Davies, C.W., and Doudna, J.A. (2014). Cas1-Cas2 complex formation mediates spacer acquisition during CRISPR-Cas adaptive immunity. Nat. Struct. Mol. Biol. *21*, 528–534.

Nuñez, J.K., Lee, A.S.Y., Engelman, A., and Doudna, J.A. (2015a). Integrase-mediated spacer acquisition during CRISPR-Cas adaptive immunity. Nature *519*, 193–198.

Nuñez, J.K., Harrington, L.B., Kranzusch, P.J., Engelman, A.N., and Doudna, J.A. (2015b). Foreign DNA capture during CRISPR-Cas adaptive immunity. Nature *527*, 535–538.

Nuñez, J.K., Bai, L., Harrington, L.B., Hinder, T.L., and Doudna, J.A. (2016). CRISPR immunological memory requires a host factor for specificity. Mol. Cell *62*, 824–833.

Poteete, A.R., Fenton, A.C., and Murphy, K.C. (1988). Modulation of Escherichia coli RecBCD activity by the bacteriophage lambda Gam and P22 Abc functions. J. Bacteriol. *170*, 2012–2021.

Punjani, A., Rubinstein, J.L., Fleet, D.J., and Brubaker, M.A. (2017). cryoSPARC: algorithms for rapid unsupervised cryo-EM structure determination. Nat. Methods *14*, 290–296.

Rollie, C., Graham, S., Rouillon, C., and White, M.F. (2018). Prespacer processing and specific integration in a Type I-A CRISPR system. Nucleic Acids Res. *46*, 1007–1020.

Samai, P., Smith, P., and Shuman, S. (2010). Structure of a CRISPR-associated protein Cas2 from Desulfovibrio vulgaris. Acta Crystallogr. Sect. F Struct. Biol. Cryst. Commun. *66*, 1552–1556.

Sapranauskas, R., Gasiunas, G., Fremaux, C., Barrangou, R., Horvath, P., and Siksnys, V. (2011). The Streptococcus thermophilus CRISPR/Cas system provides immunity in Escherichia coli. Nucleic Acids Res. *39*, 9275–9282.

Shiimori, M., Garrett, S.C., Graveley, B.R., and Terns, M.P. (2018). Cas4 nucleases define the PAM, length, and orientation of DNA fragments integrated at CRISPR loci. Mol. Cell *70*, 814–824.e6.

Sternberg, S.H., Richter, H., Charpentier, E., and Qimron, U. (2016). Adaptation in CRISPR-Cas systems. Mol. Cell *61*, 797–808.

van Heel, M., and Keegstra, W. (1981). IMAGIC: a fast, flexible and friendly image analysis software system. Ultramicroscopy *7*, 113–129.

Wang, J., Li, J., Zhao, H., Sheng, G., Wang, M., Yin, M., and Wang, Y. (2015). Structural and mechanistic basis of PAM-dependent spacer acquisition in CRISPR-Cas systems. Cell *163*, 840–853.

Wei, Y., Terns, R.M., and Terns, M.P. (2015). Cas9 function and host genome sampling in type II-A CRISPR-Cas adaptation. Genes Dev. *29*, 356–361.

Wright, A.V., and Doudna, J.A. (2016). Protecting genome integrity during CRISPR immune adaptation. Nat. Struct. Mol. Biol. *23*, 876–883.

Wright, A.V., Liu, J.-J., Knott, G.J., Doxzen, K.W., Nogales, E., and Doudna, J.A. (2017). Structures of the CRISPR genome integration complex. Science *357*, 1113–1118.

Xiao, Y., Ng, S., Nam, K.H., and Ke, A. (2017). How type II CRISPR-Cas establish immunity through Cas1-Cas2-mediated spacer integration. Nature *550*, 137–141.

Yosef, I., Goren, M.G., and Qimron, U. (2012). Proteins and DNA elements essential for the CRISPR adaptation process in Escherichia coli. Nucleic Acids Res. *40*, 5569–5576.

Zetsche, B., Gootenberg, J.S.S., Abudayyeh, O.O.O., Slaymaker, I.M.M., Makarova, K.S.S., Essletzbichler, P., Volz, S.E.E., Joung, J., van der Oost, J., Regev, A., et al. (2015). Cpf1 is a single RNA-guided endonuclease of a class 2 CRISPR-Cas system. Cell *163*, 759–771.

Zhang, K. (2016). Gctf: Real-time CTF determination and correction. J. Struct. Biol. *193*, 1–12.

Zheng, S.Q., Palovcak, E., Armache, J.-P., Verba, K.A., Cheng, Y., and Agard, D.A. (2017). MotionCor2: anisotropic correction of beam-induced motion for improved cryo-electron microscopy. Nat. Methods *14*, 331–332.

Zivanov, J., Nakane, T., Forsberg, B.O., Kimanius, D., Hagen, W.J.H., Lindahl, E., and Scheres, S.H.W. (2018). New tools for automated high-resolution cryo-EM structure determination in RELION-3. eLife *7*, e42166.

STAR★METHODS

KEY RESOURCES TABLE

REAGENT or RESOURCE	SOURCE	IDENTIFIER
Bacterial and Virus Strains		
E. coli BL21(DE3)	Novagen	69450
Chemicals, Peptides, and Recombinant Proteins		
Recombinant DNaseI (RNase free)	Roche	03539121103
T4 PNK	ThermoFisher	EK0031
Graphene Oxide	Sigma-Aldrich	763705-25ML
IPTG	ThermoFisher	R0392
Critical Commercial Assays		
NEBNext Ultra II DNA Library Prep Kit for Illumina	NEB	E7645S
Deposited Data		
Cas1Cas2Csn2 single complex bound to DNA	This work	PDB: 6QXF, EMD: 4668
Cas1Cas2Csn2 dimer complex bound to DNA	This work	PDB: 6QXT, EMD: 4670
Cas1Cas2Csn2 filament complex bound to DNA	This work	PDB: 6QY3, EMD: 4671
Oligonucleotides		
acgaggagctattggcacaacttaca (prospacer duplex forward)	Macrogen	N/A
agttgtgccaatagctcctcgtcatt (prospacer duplex reverse)	Metabion	N/A
agatatcctacgaggttttagagctgtgttgtttcgaatg gttccaaaacaaattctaaacgctaaagaggaag (CRISPR3 Leader-Repeat-Spacer duplex for *in vitro* integration reaction forward)	Metabion	N/A
cttcctctttagcgtttagaatttgttttggaaccattcgaaa caacacagctctaaaacctcgtaggatatct (CRISPR3 Leader-Repeat-Spacer duplex for *in vitro* integration reaction forward reverse)	Metabion	N/A
Recombinant DNA		
pCRISPR3 (expressing full S. thermophilus CRISPR3 loci)	Sapranauskas et al., 2011	N/A
pCas9- (Cas9 deletion, containing the rest of the CRISPR3 loci)	Sapranauskas et al., 2011	N/A
pCas2- (Cas2 deletion, containing the rest of the CRISPR3 loci)	Sapranauskas et al., 2011	N/A
pCsn2- (Csn2 deletion, containing the rest of the CRISPR3 loci)	Sapranauskas et al., 2011	N/A
pCas1Strep (expressing C-Term Strep-tagged Cas1)	This work	N/A
pCR3ΔCRISPR (CRISPR region deletion, containing the rest of CRISPR3 loci)	This work	N/A
pCR3Δtracr (tracrRNA deletion, contain the rest of CRISPR3 locus)	Karvelis et al., 2013	N/A
Software and Algorithms		
Phyre2 webserver	Kelley et al., 2015	http://www.sbg.bio.ic.ac.uk/~phyre2/html/page.cgi?id=index
Chimera	Goddard et al., 2007	https://www.cgl.ucsf.edu/chimera/
COOT	Emsley et al., 2010	https://www2.mrc-lmb.cam.ac.uk/personal/pemsley/coot/
Refmac jelly-body refinement script	Murshudov et al., 1997	https://www2.mrc-lmb.cam.ac.uk/groups/murshudov/content/em_fitting/em_fitting.html

(*Continued on next page*)

Continued

REAGENT or RESOURCE	SOURCE	IDENTIFIER
PHENIX real space refine	Afonine et al., 2018	https://www.phenix-online.org/documentation/reference/real_space_refine.html
Motioncorr	Li et al., 2013	https://cryoem.ucsf.edu/software/driftcorr.html
Motioncor2	Zheng et al., 2017	https://msg.ucsf.edu/em/software/motioncor2.html
Gctf	Zhang, 2016	https://www.mrc-lmb.cam.ac.uk/kzhang/
Gautomatch	N/A	https://www.mrc-lmb.cam.ac.uk/kzhang/
IMAGIC	van Heel and Keegstra, 1981	https://download.imagescience.de/index.html
RELION3	Zivanov et al., 2018	https://www3.mrc-lmb.cam.ac.uk/relion/index.php?title=Main_Page
cryoSPARC	Punjani et al., 2017	https://guide.cryosparc.com/install.html
GraphPad Prism 8	N/A	https://www.graphpad.com/scientific-software/prism/

CONTACT FOR REAGENT AND RESOURCE SHARING

Further information regarding the EM data should be directed to the Lead Contact, Dale Wigley (d.wigley@imperial.ac.uk). All other information and requests for resources and reagents should be directed to and will be fulfilled by the corresponding author, Virginijus Siksnys (siksnys@ibt.lt).

METHOD DETAILS

Isolation of Cas9-Cas1-Cas2-Csn2 and its derivative complexes

For purification of the Cas9-Cas1-Cas2-Csn2 complex, a Strep-tagged version of the Cas1 protein was cloned into pETDUET-1 vector using conventional restriction enzyme cloning and expressed in the *E. coli* BL21 (DE3) strain, bearing the pCas1− plasmid (Sapranauskas et al., 2011). Cells bearing both plasmids were innoculated in 4 mL of LB media and were grown at 37°C overnight in a shaking incubator. In the morning, 10mL of overnight culture was transferred to 400 mL of fresh LB media containing ampicillin (100 μg/mL), chloramphenicol (30 μg/mL). Cells were grown at 37°C to an OD_{600} of ~0.5-0.8. Then shaking was stopped and the temperature reduced to 16°C. Upon cooling, expression was induced by adding 1 mM of IPTG and shaking resumed. Cell growth was continued for 20 h.

Cells were harvested by centrifugation at 4000 g for 10 min at 4°C. Cell pellets were resuspended in lysis buffer (10 mM Tris–HCl (pH 7.5), 300 mM KCl, 1 mM EDTA, 1 mM DTT, 0.5 mM PMSF) and disrupted by sonication for 20 min with 30 s pulses. Sonications were performed in an ice bath. Cell debis was removed by centrifugation at 15000 g at 4°C. The supernatant was decanted into a separate vessel cooled on ice and loaded onto a 1 mL Strep-Trap HP column (GE Healthcare). Column was then washed with 10 column volumes of lysis buffer and subsequently eluted with 2.5 mM desthiobiotin. Elution fractions were pooled and loaded onto Superdex S200 size exclusion chromatography (prep grade XK 16/60; GE Healthcare). Eluted fractions were analyzed by SDS-PAGE. Fractions containing all four Cas proteins were pooled and dialysed against storage buffer (10 mM Tris–HCl (pH 7.5), 300 mM KCl, 1 mM EDTA, 1 mM DTT, 50% (v/v) glycerol) at 4°C overnight and stored at −20°C. Concentration of the complex was estimated by the Pierce 660nm Protein Assay (Thermo Scientific) using bovine serum albumin (BSA) as a reference.

For derivatives of CRISPR3-Cas complex lacking different components such as Cas9, Csn2, Cas2, tracrRNA or crRNA, we co-transformed *E. coli* BL21 (DE3) strain with pET-Cas1-Strep and different pCas1- plasmids, which were lacking one additional component. Everything hence followed exactly the same procedure.

DNase I treatment prior to electron microscopy

Cas9-Cas1-Cas2-Csn2 complex was mixed with 100 U of DNaseI (recombinant RNase-free, Roche) and supplemented to a total volume of 350 μL with digestion buffer: 25 mM Tris pH 7.5, 100 mM NaCl, 5 mM $MgCl_2$, 0.5 mM $CalCl_2$, 1 mM TCEP. The reaction was dialysed against 1 L of the same buffer in 3 KDa molecular weight cut-off (MWCO) dialysis cassettes (ThermoFisher) for 4 h at 4°C. The dialysed reaction was concentrated to < 50 μL (Vivaspin 500, 30 KDa MWCO, Sartorius) prior to size-exclusion chromatography (Superose 6 increase 5/150 GL) to separate out free DNA, DNaseI and aggregates (Figures S1A and S1B). The size-exclusion buffer contained: 25 mM Tris pH 7.5, 150 mM NaCl, 1 mM TCEP. Peak fractions eluting with a predicted molecular weight of 400-800 kDa were pooled and further concentrated to approximately 0.05 mg/mL and used immediately to prepare grids for cryo-electron microscopy.

Cryo-electron microscopy grid preparation and data collection

The sample was applied to C-flat 2x1 μm holey carbon grids (400 mesh) coated with graphene oxide sheets (full details of graphene oxide coating method to be published at a later date). Grids were frozen in liquid ethane using a FEI Vitrobot Mark IV, with 4 μL of

sample applied to the graphene oxide coated side of the grid followed by a 15 s wait time and 1 s blot time. The Vitrobot chamber was maintained at close to 100% humidity and 4°C. Data were collected on a Titan Krios microscope operated at 300 KV with a FEI Falcon III detector (eBIC facility, Diamond Light Source). Images were collected in integrating mode, with a nominal defocus range of −1.3 to −2.8 μm and at a nominal magnification of 75,000, which yielded a calibrated pixel size of 1.085 Å. A dose rate of 109.2 e^{-}/pixel/s was applied over a 1 s exposure, resulting in a total accumulated dose of 93 $e^{-}/Å^2$. A total of 5048 images were collected, each collected as a movie stack of 39 frames.

Data processing – Cas1-Cas2-Csn2-DNA monomer complex

Movie frames were aligned and summed using Motioncor2 (Zheng et al., 2017). Template-free particle picking in Gautomatch, using a circular diameter of 240 Å, was initially used to pick particles for the generation of templates for reference-based autopicking. Contrast transfer (CTF) parameters were estimated for individual particles on each micrograph using Gctf (Zhang, 2016). Micrographs with crystalline ice, and those with incomplete graphene oxide coverage, were excluded to leave 4908 images for further processing. Initially, a total of 853,391 particles were extracted 4x binned for two rounds of 2D classification in cryoSPARC (Punjani et al., 2017) to remove obvious artifacts and filamentous particles. This resulted in 516,595 Cas1-Cas2-Csn2 particles for 3D classification in RELION3 (Zivanov et al., 2018) with global searches and no mask applied. From this, 341,031 particles from classes showing full occupancy of all of the protein components were selected and re-extracted unbinned (Figure S2A). The starting template used for the first refinement was an *ab initio* map, generated from the data using cryoSPARC and all subsequent refinements used starting models low-pass filtered to at least 30 Å resolution with soft masks based on 12-15 Å low-pass filtered maps extended by 5-10 pixels. With D2 symmetry applied, the particles refined to a resolution of 4.0 Å in RELION3, as estimated by the 'gold-standard' 0.143 Fourier Shell Correlation (FSC) cut-off. After Bayesian polishing in RELION3 (frames 1-25, 60 $e^{-}/Å^2$ total dose), 3D refinement gave maps at 3.6 Å and 3.3 Å resolution (0.143 FSC cutoff) with C1 and D2 symmetry respectively.

Regions of the map corresponding to the Cas1 and particularly the Cas2 dimers had weaker density highlighting heterogeneity in the dataset. Therefore, 3D classification without alignment was used to separate out different states of the complex (Figure S2B). This revealed slight rotations of the protruding Cas1 dimers relative to the core of the complex, however, refining separate classes showed no difference in the complexes apart from the Cas1 dimer flexibility. Therefore, 306,470 particles, from five of the classes containing complete density for the complex, were pooled to obtain a higher resolution overall map. The final 3D refinement in RELION3 gave a map for deposition at 3.6 Å resolution with C1 symmetry and at 3.2 Å resolution (0.143 FSC cutoff) with D2 symmetry (Figure S2C). Both maps were used for figure making and sharpened with a B-factor of −80 $Å^2$. The D2-symmetrised map was used to aid model building for the protein (Figures S2E and S2F) and is deposited as a supplementary map. Even without symmetry applied, the orientations of the bound DNA are mixed as the redundant protein symmetry overshadowed the much smaller DNA signal during image alignment.

Model Building – Cas1-Cas2-Csn2-DNA monomer complex

The Phyre2 server (Kelley et al., 2015) was used to generate a homology model of StCsn2 based on the structure of SpCsn2 (PDB 3V7F). The model was rigid-body fitted into the density, followed by iterations of jelly-body refinement with Refmac (Murshudov et al., 1997) and manual rebuilding in Coot (Emsley et al., 2010). This was repeated for the corresponding adjacent chain to cover the two different StCsn2 conformations within a single dimeric unit. The previously identified Ca^{2+}-binding regions were compared to the various Csn2 crystal structures. One site per chain, occupied in all current Csn2 structures, had clear strong density that could accommodate a Ca^{2+} ion in the canonical binding position. Once complete, the two chains were copied and rigid-body-fitted into the map to generate the four symmetry-related Csn2 dimers in the complex. A similar process was used to build the two conformations of StCas1 by starting with one of the four dimers within the complex. Again, the initial template was a homology model generated by the Phyre2 server, this time from the crystal structure of EfCas1 (PDB 5XVN). After combining the eight Csn2 and eight Cas1 chains, further iterations of real space refinement with PHENIX (Afonine et al., 2018) were carried out using the D2 map before finally switching to the non-symmetrised 3.6 Å map. At this stage, 25 bp of idealized B-form linear dsDNA was rigid-body fitted into the same map. DNA sequencing revealed no sequence preference within the sample (Figures S5C), so one strand was randomly assigned as poly-dT and the other poly-dA. After a final round of real-space refinement against the 3.6 Å map (Figure S2D), the final model statistics were generated (Table 1).

Data processing – Cas1-Cas2-Csn2-DNA dimer complex

Initial 2D and 3D classification runs revealed a small population of particles corresponding to larger assemblies of Cas1-Cas2-Csn2, which were distinct from the filamentous particles observed in images of the sample (Figures S1C and S1D). After re-extracting these particles with a larger box size, cryoSPARC was used to generate an initial *ab initio* model. From this, 30 Å low-pass filtered reprojections were generated with IMAGIC (van Heel and Keegstra, 1981) for template-based autopicking with Gautomatch (Zhang, 2016). The new set of larger particles was 4x binned and then cleaned using 2D classification (cryoSPARC) to remove contamination, filamentous particles, and obvious monomer complexes until 38,473 particles remained. Following 3D refinement in RELION3, with 2x binned re-extracted particles, 3D classification without alignment isolated a homogeneous subset of particles with complete density for the complex (Figure S3A). These 9,010 particles were re-extracted unbinned and regrouped. 3D refinement in RELION3 yielded resolutions of 8.9 Å and 7.9 Å with C1 and D2 symmetry respectively (0.143 FSC cutoff). Following Bayesian polishing, the

resolution improved to 8.1 Å and 7.0 Å respectively. To improve the heterogeneous density at the middle Cas2-DNA interface, a focused 3D classification without alignment was run using a low-pass filtered mask around just the two middle Cas1Cas2 subcomplexes, the neighboring Csn2 tetramers and the DNA (Figure S3B). A homogeneous class containing 3,109 particles was selected with significantly stronger density in the masked region. A final 3D refinement in RELION3 gave the deposited map with a resolution of 8.9 Å (0.143 FSC cutoff) with C1 symmetry and a global sharpening B-factor of –250 Å^2 (Figure S3C). As with the monomer unit, the protein components were symmetrically related so a map at 7.2 Å (0.143 FSC cutoff) resolution was generated with D2 symmetry to aid model fitting and interpretation (Figure S3E).

Model Building – Cas1-Cas2-Csn2-DNA dimer complex

The coordinates of the monomeric Cas1$_8$-Cas2$_4$-Csn2$_8$ complex were rigid-body fitted (Coot) at each end of the D2-symmetrised map to give a dimeric assembly on DNA. Between each set of coordinates there was also additional density that could accommodate an additional two sets of Cas1$_4$Cas2$_2$ subcomplexes; four Cas1 dimers and two Cas2 dimers. The coordinates for each additional Cas1 dimer, complexed with the C-terminal β strand of Cas2, were taken from the monomeric Cas1-Cas2-Csn2 model and rigid-body fitted into density. The density for the dimeric, globular Cas2 N-termini was sufficiently ordered to dock Cas2 for the first time in the context of these complexes (Figures S3B and S3E). A homology model was generated for a Cas2 monomer using Phyre2, based on the crystal structure of SpCas2 (PDB 5ZYF), duplicated and each chain was rigid-body fitted into the map. This dimer was then duplicated, rigid-body fitted into the second free position, and could also be docked into the Cas2 positions within the monomeric Cas1-Cas2-Csn2 units within the dimer complex. The two fragments of idealized, linear dsDNA within each Cas1-Cas2-Csn2 unit were rigid-body fitted and extended by two residues toward the middle Cas2 dimers. At this resolution, it is not clear what happens to the DNA at this interface, but it is certain that the path of the two dsDNA ends would be obstructed by the two interacting Cas2 dimers. Due to the limited resolution all residues were clipped at the C_α position. REFMAC jelly-body and then PHENIX rigid-body refinement was performed against the C1 map to generate final model statistics (Figure S3D) (Table 1).

Data processing – Cas1-Cas2-Csn2-DNA filament complexes

Filament particle classes from initial 2D runs were used as templates in Gautomatch to pick a particle set with a size aiming to pick units of the filaments with similar length to the Cas1-Cas2-Csn2 dimer complex. A total of 349,849 particles were extracted, 2x binned and cleaned with multiple rounds of 2D classification in cryoSPARC. Different orientations were classified separately to filter out distinct Cas1-Cas2-Csn2 monomer and dimer particles to leave a combined particle set of 66,576 particles. An *ab initio* 3D model was generated from the particles and then 3D refined to a resolution of 9.1 Å (0.143 FSC cutoff) with C1 symmetry, both in cryoSPARC. From this, unmasked 3D classification in RELION3 without alignment produced related filament classes of different lengths, the minimum unit corresponding to a similar size as the Cas1-Cas2-Csn2 dimer complex (Figure S7A). All particles were unbinned and Bayesian polished (RELION3). 3D refinement (RELION3) with a mask around the minimal filament unit gave a map at 7.7 Å with D2 symmetry respectively (Figure S7B). This map was used to aid model docking and for figures comparing the arrangement of the complex with that of the Cas1-Cas2-Csn2 dimer complex (Figures 5 and 6). A final map for deposition, at 9.1 Å resolution with C1 symmetry, was generated from a 3D refinement (RELION3) with an extended mask (Figure S7B).

Model Building – Cas1-Cas2-Csn2-DNA filament complexes

The Cas1-Cas2-Csn2 filament model was generated in a similar way to that of the dimer complex with the minimal unit masked map used to initially rigid body fit components. Only the coordinates for the minimal observed filament unit were docked and comprised the same relative numbers of the subunits as the dimeric complex but arranged in a different assembly. For this complex, the Cas2 dimers were as well ordered as in the dimer complex and there was not sufficient resolution to unequivocally dock their position. As a result, Cas2 dimers were excluded from the deposited model but were docked in the best fitting orientation for figure making for comparing their relative position to those in the dimer complex (Figures 5 and 6). Also, in contrast to the dimer structure, the dsDNA density was clearly continuous throughout the entire length of the filament (Figure S7D) and so linear idealized dsDNA was rigid-body fitted based on the position assigned in the monomeric structure. The DNA density was observed to bend slightly so REFMAC jelly-body refinement was carried out for just the DNA chains. All residues were clipped at the C_α position and refinement was limited to rigid body-fitting of structural groups in Phenix against the deposited 9.1 Å map to generate final model statistics (Figure S7C) (Table 1).

Sample library preparation for deep sequencing analysis

Cas9-Cas1-Cas2-Csn2 complexes before and after DnaseI treatment were mixed with equal volume of Phenol:Chloroform:Isoamyl alcohol mixture (Roth) and thoroughly mixed and briefly centrifuged. Upper fraction was carefully removed and transferred to a new tube. To this DNA containing fraction, 3 M Sodium acetate was added to a final concentration of 300 mM, 1 μl of 24% w/v PEG 6000 and 2.5 volumes of 95% ethanol chilled to 4°C. Contents were thoroughly mixed by pipetting up and down and left to precipitated overnight at 4°C. Precipitation mixture was centrifuged at 4°C for 1 h on a microcentrifuge at ~14000 g. The liquid was decanted and the remainder was removed carefully with a pipette without disturbing the pellet. Pellet was then gently washed with ice cold 70% ethanol and centrifuged at 4°C for 30 min on a microcentrifuge at ~14000 g. The ethanol was removed carefully and the pellet was

then air-dried for 15 min and subsequently resuspended in 50 μl of TE buffer (10 mM Tris-HCl pH 8, 1 mM EDTA). Concentration was determined using a NanoDrop device (Thermo Fisher Scientific).

Samples for deep sequencing were prepared using NEBNext Ultra II DNA Library Prep Kit for Illumina (NEB) according to manufacturer's instructions. Briefly, DNA ends were prepared for adaptor ligation using NEBNext Ultra II End Prep Enzyme Mix. Afterward, adapters were ligated using NEBNext Ultra II Ligation Master Mix and opened up using USER enzyme. DNA was gel purified and then barcoded and amplified via PCR.

PCR products were again gel purified. Concentrations were determined using both NanoDrop and Qubit (Thermo Fisher Scientific). Samples were sent for MiSeq sequencing and analysis (Figures S5A–S5C).

Integration reactions into linear substrates

75 nM of Cas9-Cas1-Cas2-Csn2, Cas1-Cas2-Csn2 or Cas1-Cas2 protein complexes were incubated with 50 nM of a synthetic linear oligoduplex imitating part of the CRISPR region (Figure S5D) and 250 nM pre-spacer oligoduplex (in the case of Cas1-Cas2 complex) in the integration buffer (20 mM HEPES-NaOH, pH 7.5, 25 mM KCl, 10 mM $MgCl_2$, 1 mM DTT, 10% V/V DMSO). Reactions were stopped by adding an equal volume of a phenol:chlorophorm:isoamyl alcohol mixture. Samples were thoroughly mixed and briefly centrifuged. The aqueous phase was carefully removed to a separate tube and mixed with denaturing dye solution (100% formamide, 0.025% bromphenol blue). Samples were then heated for 5 min at 95°C and upon cooling, loaded onto a denaturing polyacrylamide gel containing 8 M urea (Figures S5E and S5F). After electrophoresis, the gel was dried and exposed to a phosphorescent screen (FujiFilm). Radioactive bands were visualized on a FujiFilm FLA-5100 scanner.

Linear partial CRISPR region substrate was prepared by annealing the following oligonucleotides (Metabion):

agatatcctacgaggttttagagctgtgttgtttcgaatggttccaaaacaaattctaaacgctaaagaggaag
cttcctctttagcgtttagaatttgttttggaaccattcgaaacaacacagctctaaaacctcgtaggatatct

Pre-spacer oligoduplex was made by annealing following oligonucleotides (Metabion):

acgaggagctattggcacaacttaca
agttgtgccaatagctcctcgtcatt

Linear CRISPR region substrates were prepared as follows. Oligonucleotides were radioactivelly labeled on the 5'-end with γ^{32}ATP (Perkin Elmer) by using standard Polynucleotide Kinase (Thermo Fisher Scientific) reaction. On the 3'-end, labeling was performed using Terminal Deoxynucleotidyl Transferase (Thermo Fisher Scientific) and α^{32}Cordycepin triphosphate (Perkin Elmer). Substrates labeled on the 3′ end were purified by phenol:chlorophorm:isoamyl alcohol extraction and ethanol precipitation, because TdT reaction buffers contain Co^{2+} ions, which may interfere with the downstream reaction. Only one oligonucleotide of the pair was labeled at a time. Afterward, labeled and non-labeled oligonucleotides were mixed together and annealed by heating to 95°C in a water bath and then cooling to room temperature.

DATA AND SOFTWARE AVAILABILITY

The accession numbers for the deposited coordinates and maps from this work are:

PDB 6QXF and EMD-4668 (Cas1-Cas2-Csn2-DNA monomer complex), PDB 6QXT and EMD-4670 (Cas1-Cas2-Csn2-DNA dimer complex), PDB 6QY3 and EMD-4671 (Cas1-Cas2-Csn2-DNA filament complex).

Molecular Cell, Volume *75*

Supplemental Information

Structure of the DNA-Bound Spacer Capture Complex of a Type II CRISPR-Cas System

Martin Wilkinson, Gediminas Drabavicius, Arunas Silanskas, Giedrius Gasiunas, Virginijus Siksnys, and Dale B. Wigley

Figure S1: Cryo-EM images of the mixture. Related to Figure 1, Figure 2 and Figure 5.

Figure S2: EM processing for the Cas1Cas2Csn2 monomer complex. Related to Figure 2.

Figure S3: EM processing for the Cas1Cas2Csn2 dimer complex. Related to Figure 5.

Figure S4: DNA binds within a positive channel through the Csn2 tetramers. Related to Figure 2.

Figure S5: Deep-sequencing analysis of DNA captured by Cas1- Cas2-Csn2-Cas9 complex and integration assays. Related to Figure 1, Figure 2 and Figure 5.

Figure S6: *In vitro* interactions of CRISPR3-Cas proteins. Related to Figure 1.

Figure S7: EM processing for the Cas1Cas2Csn2 filament complex. Related to Figure 5.

Figure S1. Related to Figure 1, Figure 2 and Figure 5.

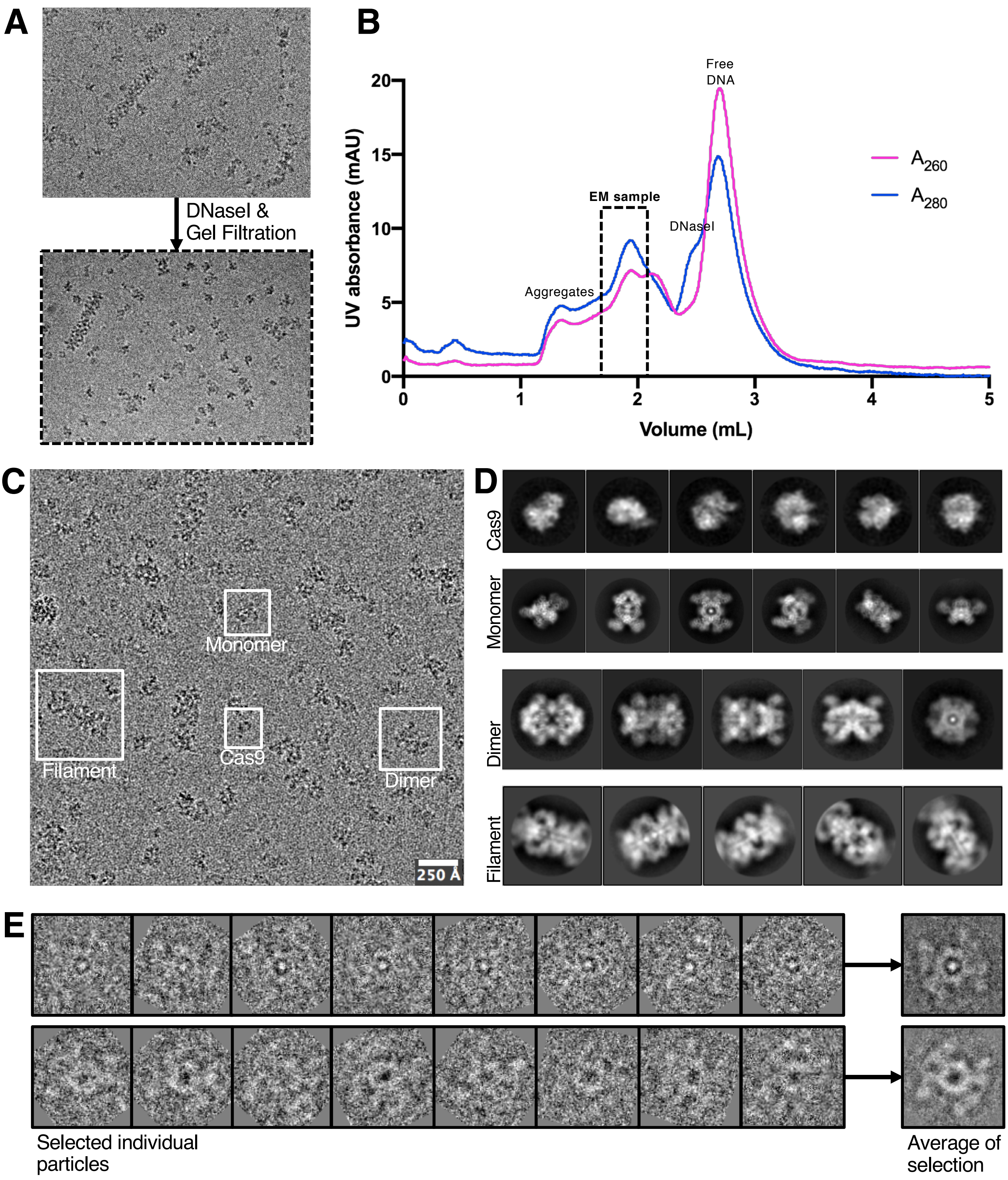

Figure S1. Cryo-EM images of the mixture. Related to Figure 1, Figure 2 and Figure 5.

(A) Initial cryo image of the sample showing long threads of DNA.

(B) Cryo image after DNaseI treatment. Both images were taken on a Technai T12 microscope with CCD detector.

(C) Section of a micrograph from the Krios-Falcon III dataset. An example of each of the different particle populations is boxed.

(D) Representative 2D classes generated in RELION from the Krios-Falcon III dataset showing different views for each of the four particle populations.

(E) A selection of particles from a subset of the Krios-Falcon III dataset that represented just the front view of the monomer complex. The particles could be separated into two species: 98.6 % were DNA bound (top row) and 1.4 % were fully assembled without DNA (bottom row). Particles and class averages were generated and displayed using Relion.

Figure S2. Related to Figure 2.

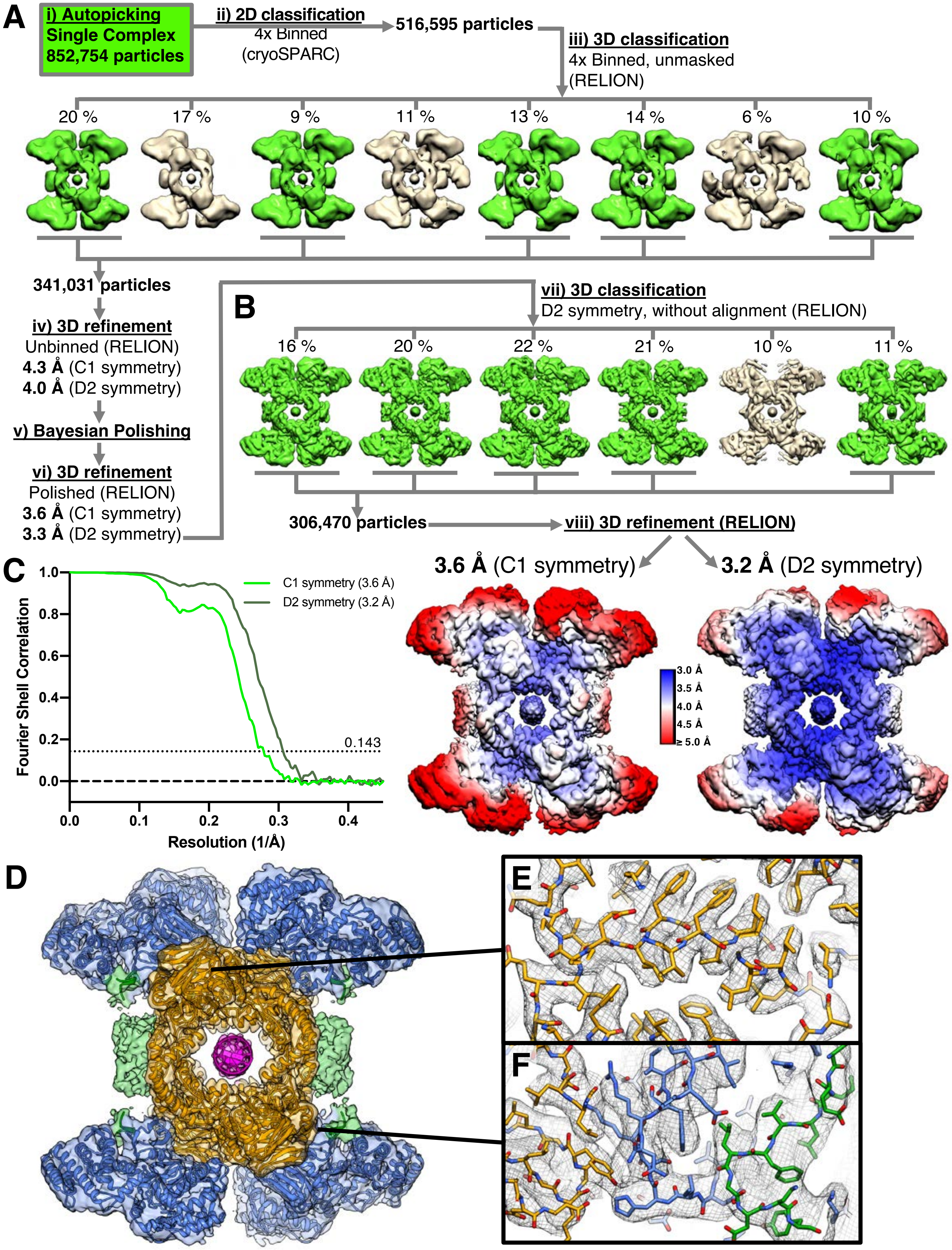

Figure S2. EM processing for the Cas1Cas2Csn2 monomer complex. Related to Figure 2.

(A) Scheme overview of the initial processing steps for the monomer particle set.

(B) The 3D classes from the final classification step are shown. The classes show flexibility in the orientation of the Cas1 dimers relative to the rest of the complex.

(C) FSC plot for the final monomer maps (left). The deposited C1 and supplemental D2-symmetrised maps are shown coloured by local resolution (calculated using RELION) (right).

(D) The deposited 3.6 Å monomer map is shown as a transparent surface with the coordinates underneath. Both are coloured similarly to Figure 2.

(E) A section of the 3.2 Å D2-symmetrised map displayed as grey mesh showing ordered side-chain density for the selected region within Csn2.

(F) A section of the 3.2 Å D2-symmetrised map displayed as grey mesh showing the fit of the contact regions between the C-terminus of Cas2, the Cas1 NTD and the Csn2 head domain.

Figure S3. Related to Figure 5.

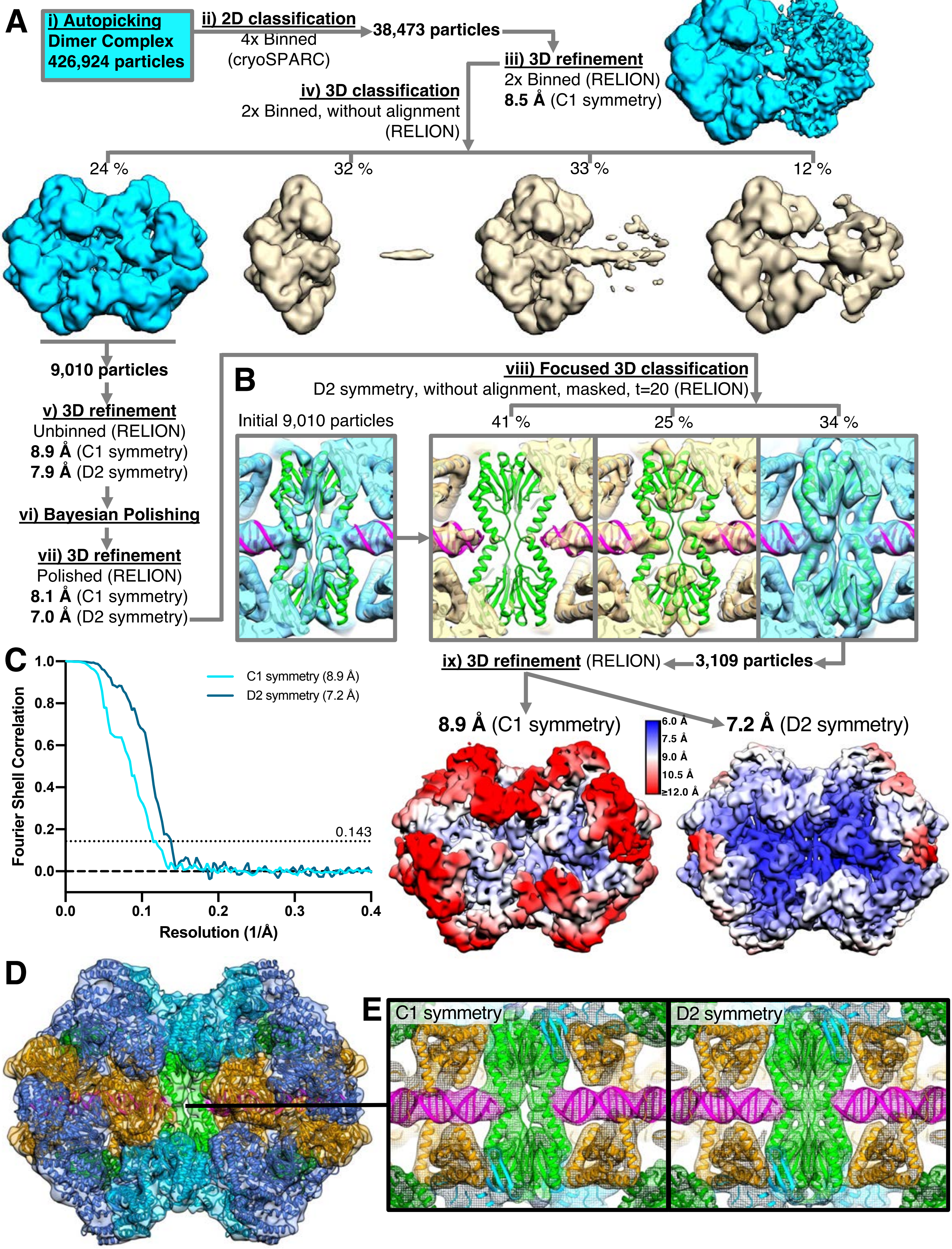

Figure S3. EM processing for the Cas1Cas2Csn2 dimer complex. Related to Figure 5.

(A) Scheme overview of the initial processing steps for the dimer particle set.

(B) A section around the middle Cas2 dimer,s which cap the DNA fragment ends, are shown for each of the 3D classes from the final focused classification step as well as for the initial map before classification. The classes show flexibility in the orientation of the Cas1 dimers relative to the rest of the complex. A homogeneous class with strong density for the region was selected for the final refinement.

(C) FSC plot for the final dimer maps (left). The deposited C1 and supplemental D2-symmetrised maps are shown coloured by local resolution (calculated using RELION) (right).

(D) The deposited 8.9 Å dimer map is shown as a transparent surface with the coordinates underneath. Both are coloured similarly to Figure 5.

(E) A section of both the C1 and D2-symmetrised maps is displayed as coloured mesh showing the fit of the ordered Cas2 dimers capping the end of the DNA fragments. There is no free density to suggest that the DNA ends are joined.

Figure S4. Related to Figure 2.

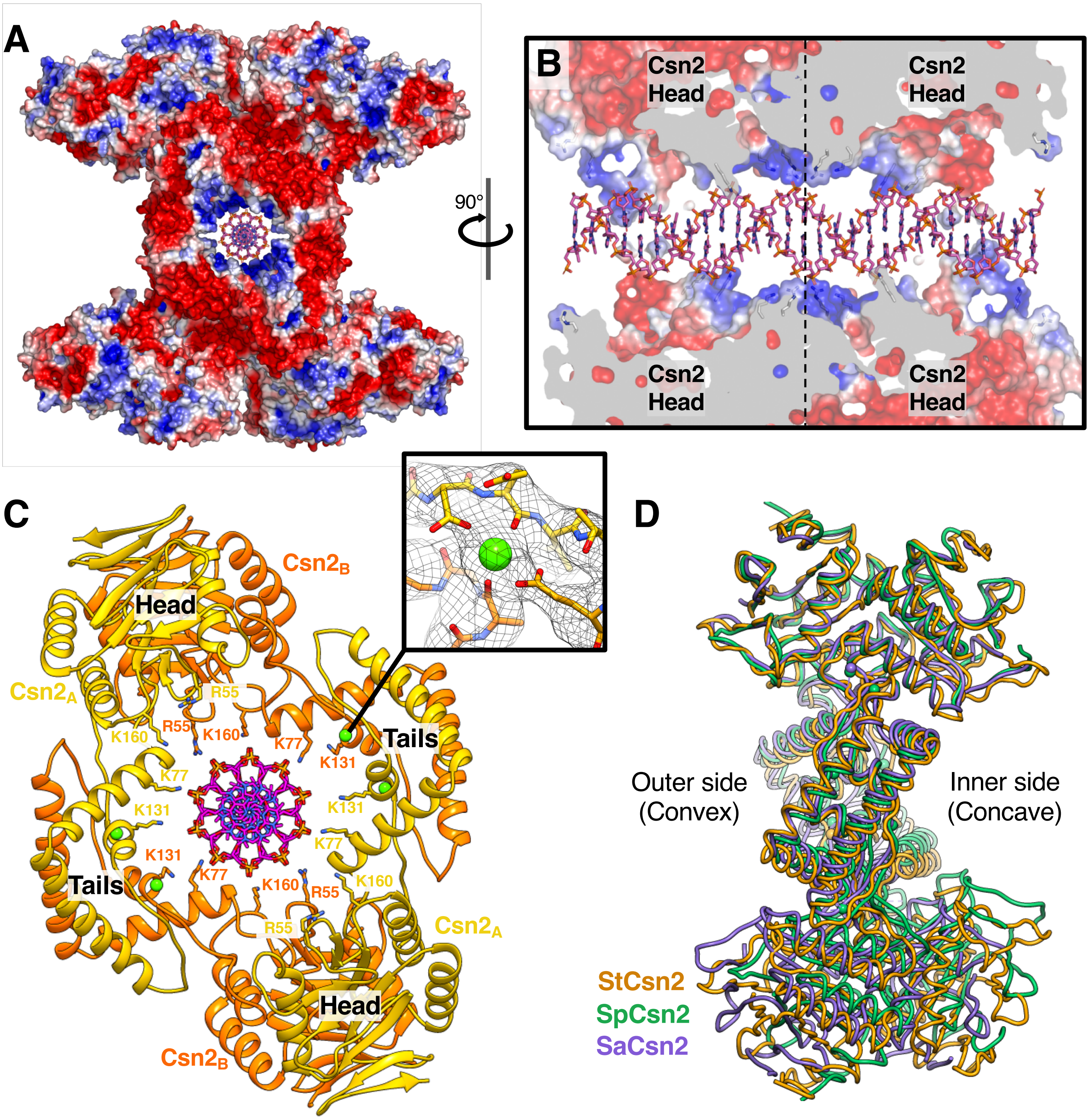

Figure S4. DNA binds within a positive channel through the Csn2 tetramers. Related to Figure 2.

(A) Overall charge distribution on the surface of the monomer structure calculated by APBS displaying a range from -3 (red) to +3 (blue) kT/e. The bound DNA was deleted from the charge calculation but overlaid for reference.

(B) Cutaway showing the charge distribution along the central channel through the two Csn2 tetramers, which are divided here by a dotted line. Due to the concave nature of the interacting Csn2 tetramer surfaces, more DNA contacts are found in the centre of the channel than in the peripheries.

(C) A single Csn2 tetramer is displayed as cartoon coloured by chain showing the conserved residues positioned to bind non-specifically to the DNA backbone. Bound Ca2+ ions are shown as green spheres.

(D) Side view with the crystal structures of different Csn2 tetramers (Koo et al., 2012; PDB 3TOC and Ellinger et al., 2012; PDB 3QHQ) aligned to the top head domain of a Csn2 tetramer from the complexes described in this work. The concave side formed by different conformations of the Csn2A and Csn2B chains is conserved in all three structures but with varying degrees of rotation.

Figure S5. Related to Figure 1, Figure 2 and Figure 5

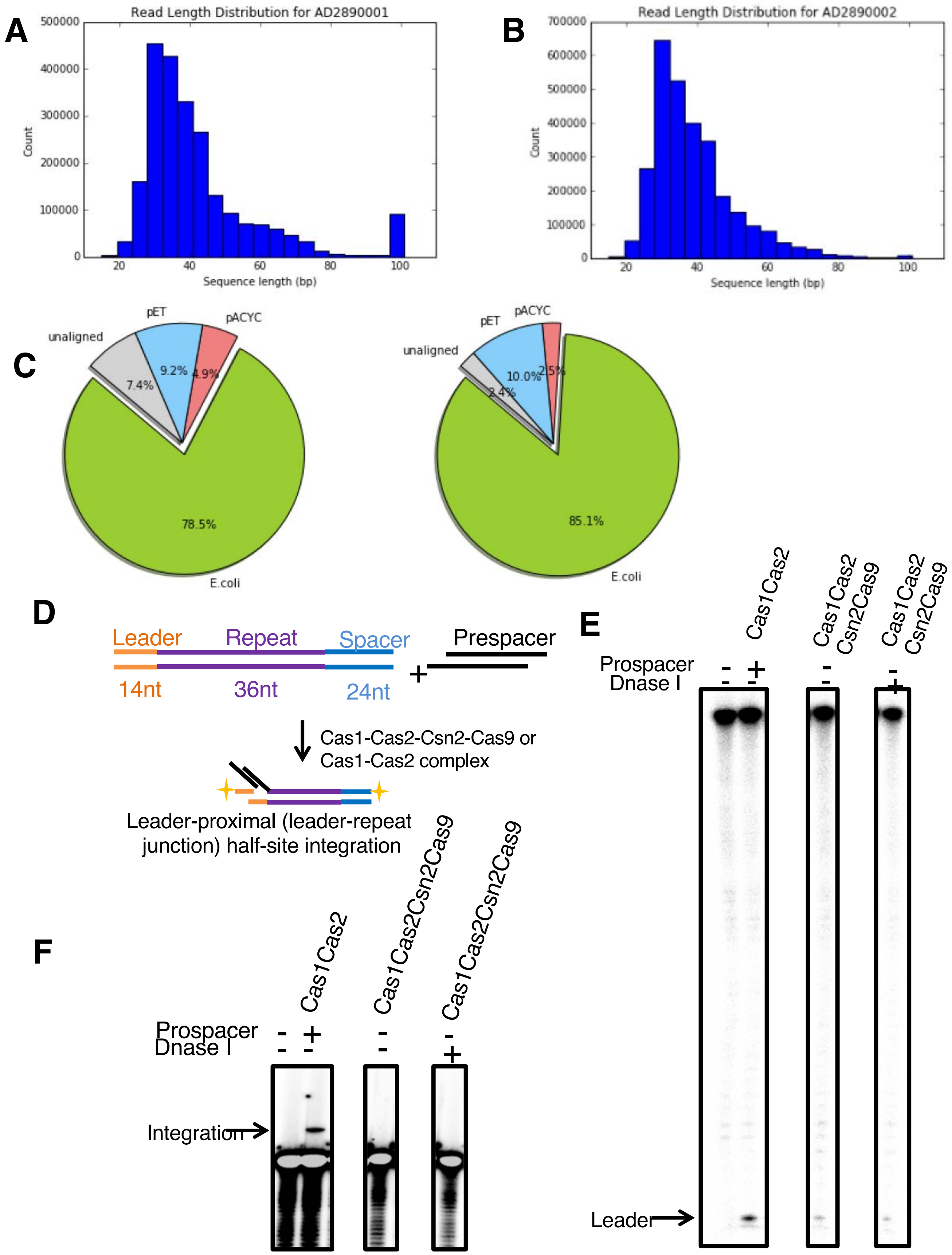

Figure S5. Deep-sequencing analysis of DNA captured by Cas1- Cas2-Csn2-Cas9 complex and integration assays. Related to Figure 1, Figure 2 and Figure 5.

(A) DNA captured by Cas1-Cas2-Csn2-Cas9 length distribution before DNAse I treatment. Many reads of 100 or more bp are evident. They are the likely filamentous structures seen in initial negative stain EM.

(B) DNA captured by Cas1-Cas2-Csn2-Cas9 length distribution after DNAse I treatment. The number of reads of 100+ length is decreased dramatically and the main distribution is more streamlined. This is consistent with the homogenization of DNA in the sample.

(C) Maps of the DNA captured by Cas1-Cas2-Csn2-Cas9 complexes onto *E. coli* genome and plasmids encoding CRISPR3-Cas system. As expected, DNA distribution closely matched expected DNA concentrations in the cell. No enrichment near PAM sequences was detected.

(D) Schematic representation of the integration assay. Prespacer was not added in case of Cas1-Cas2-Csn2-Cas9, as it was assumed that DNA bound by the complex may serve as one. 28

(E) Integration assay into substrates with labelled 5' end. Both complexes catalyse the nicking at the Leader-Repeat junction as evidenced by the release 14nt Leader fragment indicative of integration.

(F) Integration assay into substrates with labelled 3' ends. Only Cas1-Cas2 complex, but not the full complex exhibited permanent integrations resulting in the elongation of the initial substrate strand.

Figure S6. Related to Figure 1.

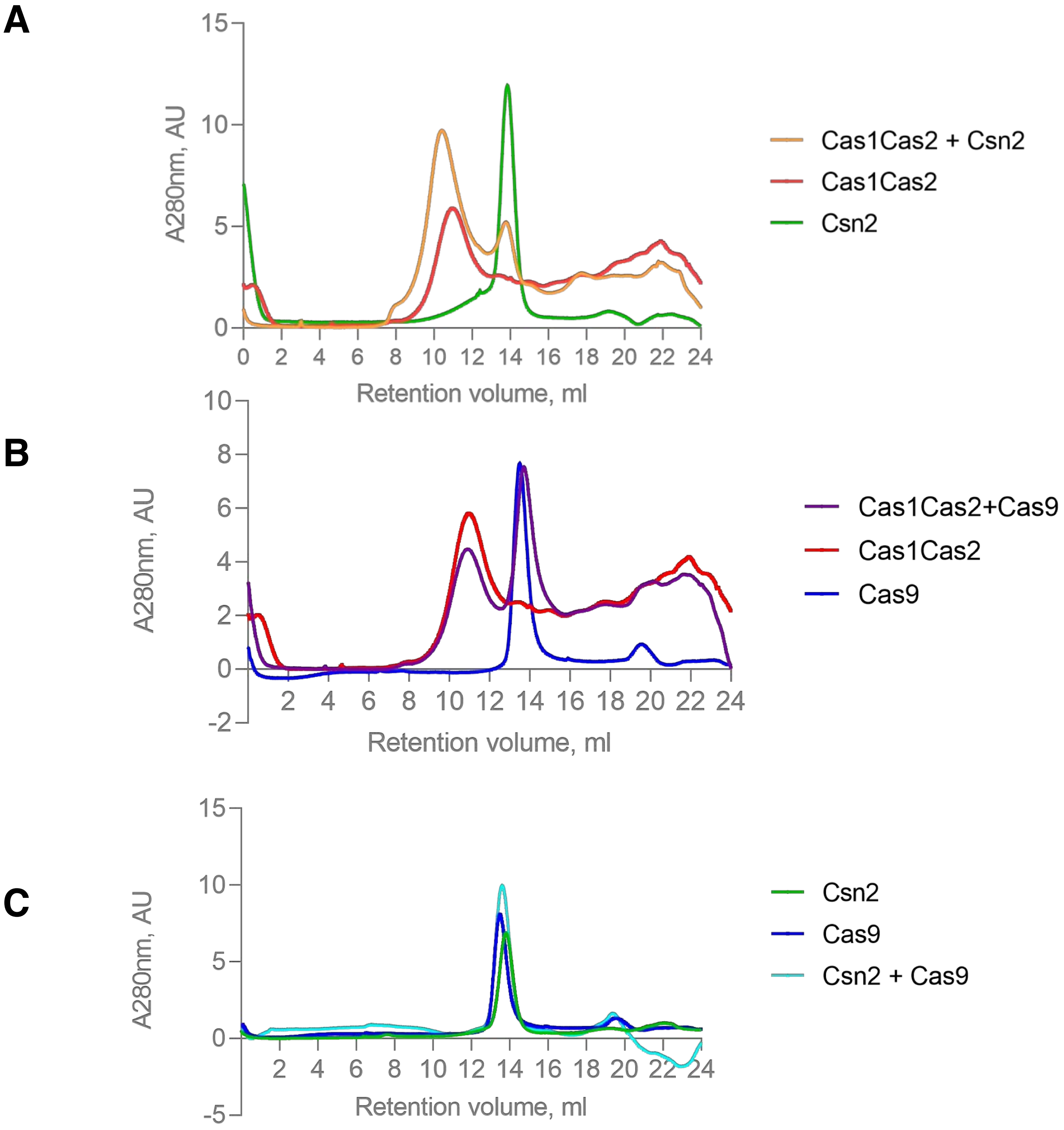

Figure S6. *In vitro* interactions of CRISPR3-Cas proteins. Related to Figure 1.
(A) Cas1-Cas2 interacts with Csn2 directly. Cas1-Cas2 eluted in the void volume, because they Had bound nucleic acids. However, upon mixing it with excess Csn2, a portion of Csn2 moved to the void volume as well.
(B) Cas1-Cas2 does not directly interact with Cas9. Cas9 peak neither decreased in size not moved to the left, therefore Cas9 did not interact with Cas1-Cas2.
(C) Csn2 does not interact with Cas9 directly, as the retention volume of the combined proteins did not shift left. Cas9 used for these experiments was complexed with tracr:crRNA duplex.

Figure S7. Related to Figure 5.

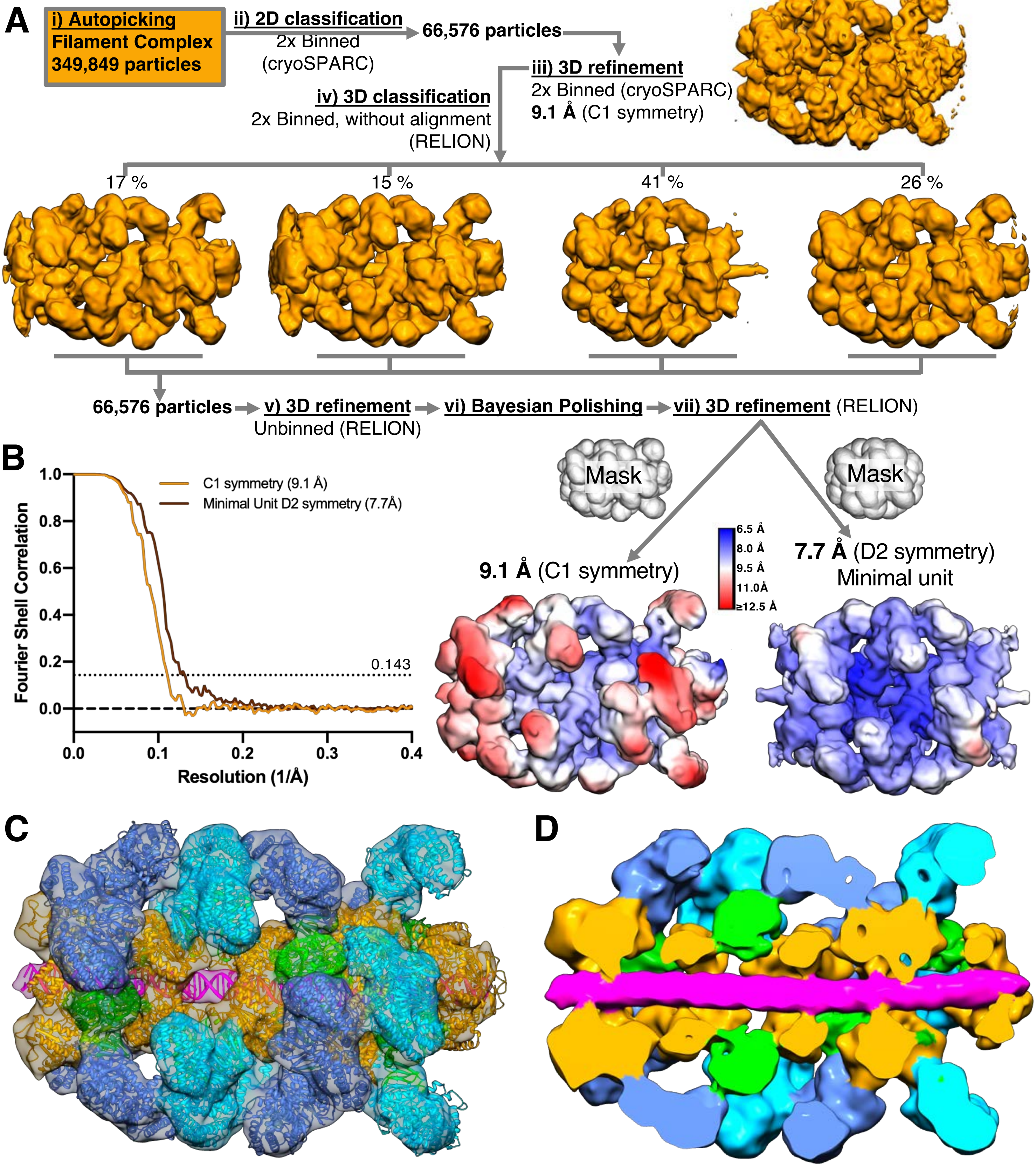

Figure S7. EM processing for the Cas1Cas2Csn2 filament complex. Related to Figure 5.

(A) Scheme overview of the initail processing steps for the filament particle set.

(B) FSC plot for the final filament maps (left). The deposited C1 and supplemental D2-symmetrised maps, coloured by local resolution (calculated using RELION), are displayed along with the corresponding masks used during refinement (right).

(C) The deposited 9.1 Å filament map is shown as a transparent surface with the coordinates underneath. Both are coloured similarly to Figure 5.

(D) Similar view to S7C of the deposited filament map, which is sliced in half to show the continuous density for dsDNA (magenta) through the entire filament.
